# Genome-Wide Identification and Analysis of the Polycomb Group Family in *Medicago truncatula*

**DOI:** 10.3390/ijms22147537

**Published:** 2021-07-14

**Authors:** Yuanyuan Zhao, Junchao Zhang, Zhanmin Sun, Yixiong Tang, Yanmin Wu

**Affiliations:** 1 State Key Laboratory of Grassland Agro-Ecosystems, Key Laboratory of Grassland Livestock Industry Innovation, Ministry of Agriculture, College of Pastoral Agriculture Science and Technology, Lanzhou University, Lanzhou 730020, China; zhaoyy17@lzu.edu.cn; 2 Biotechnology Research Institute, Chinese Academy of Agricultural Sciences, Beijing 100081, China; sunzhanmin@caas.cn (Z.S.); tangyixiong@caas.cn (Y.T.); 3 College of Qinghai-Tibetan Plateau, Southwest Minzu University, Chengdu 610041, China; jczhang@swun.edu.cn

**Keywords:** PcG family, *Medicago truncatula*, genome-wide, gene structure, evolutionary relationship, expression pattern

## Abstract

Polycomb group (PcG) proteins, which are important epigenetic regulators, play essential roles in the regulatory networks involved in plant growth, development, and environmental stress responses. Currently, as far as we know, no comprehensive and systematic study has been carried out on the PcG family in *Medicago truncatula*. In the present study, we identified 64 PcG genes with distinct gene structures from the *M. truncatula* genome. All of the PcG genes were distributed unevenly over eight chromosomes, of which 26 genes underwent gene duplication. The prediction of protein interaction network indicated that 34 *M. truncatula* PcG proteins exhibited protein–protein interactions, and MtMSI1;4 and MtVRN2 had the largest number of protein–protein interactions. Based on phylogenetic analysis, we divided 375 PcG proteins from 27 species into three groups and nine subgroups. Group I and Group III were composed of five components from the PRC1 complex, and Group II was composed of four components from the PRC2 complex. Additionally, we found that seven PcG proteins in *M. truncatula* were closely related to the corresponding proteins of *Cicer arietinum*. Syntenic analysis revealed that PcG proteins had evolved more conservatively in dicots than in monocots. *M. truncatula* had the most collinearity relationships with *Glycine max* (36 genes), while collinearity with three monocots was rare (eight genes). The analysis of various types of expression data suggested that PcG genes were involved in the regulation and response process of *M. truncatula* in multiple developmental stages, in different tissues, and for various environmental stimuli. Meanwhile, many differentially expressed genes (DEGs) were identified in the RNA-seq data, which had potential research value in further studies on gene function verification. These findings provide novel and detailed information on the *M. truncatula* PcG family, and in the future it would be helpful to carry out related research on the PcG family in other legumes.

## 1. Introduction

A large number of studies have shown that epigenetics plays an important regulatory role throughout the life cycle from plant growth and development to senescence and death. This includes effects on the morphological architecture, physiological process, metabolic pathways, and evolutionary adaptation of plants. In recent years, the growth environment of plants has produced more abiotic stresses (such as high/low temperature, drought/water-logging, saline/alkaline stress, etc.) due to the impact of global climate change. Recent advances have suggested that epigenetic mechanisms are critical for the regulation of plant responses to stress [1]; these mechanisms include DNA methylation, histone modification, RNA methylation, chromatin remodeling, and non-coding RNA, etc.

The polycomb group (PcG), as a highly conserved epigenetic modifying protein complex, has been widely found in plants and animals and could continuously and stably inhibit gene transcription [2]. PcG proteins were first identified in *Drosophila*, which is involved in regulating the expression of homeobox genes and the development of body segments [3]. Numerous PcG proteins have been identified in several plants, including *Arabidopsis thaliana*, *Oryza sativa*, *Zea mays*, mosses, and green algae in recent years [2]. Additionally, these studies mainly focus on the regulation of important biological processes, such as plant flowering [4,5], growth and development [6,7,8], and stress responses [9,10,11]. Previous reports have indicated that 5% of genes in vertebrates and *Drosophila* [12], and 15% of genes in *A. thaliana* may be regulated by PcG proteins [13]. The PcG proteins in plants usually cannot recognize the target DNA sequence as they lack the sequence specificity. Therefore, it is necessary to localize the target gene through the guidance of specific DNA binding proteins and long non-coding RNAs, and that process will initiate correct spatiotemporal silencing [14]. PcG proteins have important biological functions in developmental transitions throughout the entire life cycle of plants, including breaking seed dormancy, the transition from vegetative growth to reproductive growth, vernalization, gametophyte and seed development, plant hormone signal transduction, response to environmental stimuli [2,15], etc. As regulators of epigenetic gene expression, PcG proteins usually perform epigenetic gene repression through the combined activity of two multi-protein complexes, polycomb-repressive complex 1 (PRC1) and polycomb-repressive complex 2 (PRC2). The functions of PRC1 differ between plants and animals [16]; this protein complex catalyzes histone H2A ubiquitination and recognizes the site of histone H3 lysine 27 trimethylation (H3K27me3) to suppress gene expression [17]. The PRC2 complex is composed of four subunits with histone methyltransferase activity and can induce H3K27me3, which results in the modification of the chromatin structure and long-lasting gene repression [18]. It was initially considered that PRC1 acts strictly downstream of PRC2, whereas recent studies suggest that the two complexes may function together or independently in transcriptional repression [19].

The core of the PRC1 complex in *Drosophila* is composed of Polycomb (Pc), Posterior sex combs (Psc), Polyhomeotic (Ph), and dRING1 proteins [20]. The PRC1 complex of plants is considered to contain five components, of which LIKE HETEROCHRO-MATIN PROTEIN 1 (LHP1, also known as TFL2) is the earliest recognized PRC1. Additionally, this component has an N-terminal CHROMO domain and a C-terminal CHROMOSHADOW domain [21]. LHP1 is localized in euchromatin and can combine with H3K27me3 to participate in the repression of specific PcG target genes, which is similar to the Pc protein in animals [20]. The *lhp1* mutants of *A. thaliana* cause the misexpression of several PcG target genes involved in flower development, as well as producing morphological changes such as dwarf plants [22]. The RING-finger protein has the most conserved structure, consisting of the N-terminus RING domain and the C-terminus ubiquitin-like (RAWUL) domain. Moreover, the RING-finger protein is divided into RING1 and BMI1 according to its sequence similarity. It has E3 ligase activity and can catalyze histone H2A lysine 119 monoubiquitylation (H2K119Ub1) [21]. In *A. thaliana*, RING1 is homologous to the dRING1 of *Drosophila*, which includes RING1a and RING1b, while BMI1 is homologous to the Psc of *Drosophila*, which includes AtBMI1a, AtBMI1b, and AtBMI1c [16]. EMBRYONIC FLOWER 1 (EMF1) is a plant-specific DNA binding protein with poor conservation owing to the lack of conserved motifs and annotated domains, but no homologous proteins with known functions have been found in other species [20,23]. EMF1 is located in the genomic region where H3K27me3 is modified, and this protein is necessary for the suppression of PcG target genes and other genes [17]. Vernalization1 (VRN1), as a newly added component of PRC1, is also a plant-specific protein and only exists in dicotyledons. This protein binds DNA in a non-sequence-specific manner and can inhibit FLC, the main target of the vernalization pathway [20,24].

The PRC2 complex in *Drosophila* is composed of the Enhancer of zeste (E(z)), Suppressor of zeste 12 (Su(z)12), Extra sex combs (Esc), and Nucleosome remodeling factor 55 (Nurf55) [25]. CURLY LEAF (CLF), SWINGER (SWN), and MEDEA (MEA) in *A. thaliana* are homologous to the E(z) protein in *Drosophila* [26,27,28]. Among them, CLF inhibits the expression of the flower homologous gene AGAMOUS (AG) and the Class I KNOX gene SHOOT MERISTEMLESS (STM) through H3K27me3 to control the morphology of leaves and flowers and the flowering time [29,30]. Meanwhile, CLF also plays an important role in controlling the fate of floral meristems and maintaining the activity of root meristems [31,32]. As an H3K27 methyltransferase, the binding genes of SWN are almost the same as those in CLF; thus, SWN and CLF have redundant functions [33,34]. MEA is a plant imprinted gene that is essential for seed development [35]. EMBRYONIC FLOWER 2 (EMF2) [4], VERNALIZATION 2 (VRN2) [36], and FERTILIZATION INDEPENDENT SEED 2 (FIS2) [37] are the homologous proteins of Su(z)12 in *Drosophila*. EMF2 is critical for plant growth and vegetative development [38]. The lack of functional mutant *emf2* would bypass the growth of rosette shoots and result in early flowering [4]. VRN2 is the main repressor of flowering; it is regulated by a low temperature and photoperiod and takes part in the suppression of FLC after vernalization [39,40]. Furthermore, this protein has a negative regulatory effect on flowering time in the vernalization pathway of cereal crops [41]. FIS2 can inhibit seed development without fertilization [42], and the mutants of FIS2 develop into seed-like structures in the absence of fertilization. Nevertheless, if fertilization occurs, the seed will have proliferation defects and abortion in the embryo and endosperm [43]. VRN2, FIS2, and EMF2 all have a C-terminal conserved domain containing 150 amino acids, which are called VEFS and C2H2 zinc fingers. It is noted that FIS2 contains repetitive sequences of 17 and 21 amino acids, while such repetitive sequences do not exist in VRN2 and EMF2 [44]. The PRC2 of *A. thaliana* also contains two WD40 motif proteins: FERTILIZATION-INDEPENDENT ENDOSPERM (FIE) and MULTICOPY SUPRESSOR OF IRA 1-5 (MSI1-5), which are the homologous proteins of Esc and Nurf55 in *Drosophila*, respectively [45,46]. The FIE protein encoding the tryptophan-aspartate (WD) domain represses homologous genes by interacting with other polycomb proteins and inhibits endosperm development before fertilization [47]. MSI1 is considered to be a new flowering-time gene whose function is independent of FLC [48]. In addition, numerous reports have found that these PRC2 members formed a unique complex in *A. thaliana*, mainly including FIS-PRC2, VRN-PRC2, and EMF-PRC2. Among them, FIS-PRC2 is composed of MEA, FIE, FIS2, and MSI1 [49] and has an impact on the formation of gametophytes and early seeds [2]. VRN-PRC2 is composed of CLF/SWN, FIE, VRN2, and MSI1 [50] and mainly affects flowering time through vernalization [36,51,52,53]. EMF-PRC2 is composed of CLF/SWN, FIE, EMF2, and MSI1 [50]; suppresses the premature transition from vegetative to reproductive development; and as well as participates in the regulation of floral organ development [27,54,55].

*M. truncatula* is an annual, self-pollinated diploid leguminous plant that is used as a model species for legume genetics research owing to the advantages of the small genome, short growth period, and high genetic transformation efficiency [56]. The whole-genome sequencing of *M. truncatula* has been completed, providing a major foundation for the comprehensive analysis of the evolutionary relationship and gene function of the gene family in *M. truncatula* through bioinformatics methods. Currently, there are few studies on the genome-wide identification, evolution, and functional analysis of the PcG family in *M. truncatula*. Although there are reports that several PcG genes have been identified from the *M. truncatula* genome, the analysis of the identified genes has not been comprehensive and systematic [57,58,59,60]. Accordingly, to better understand the gene structure, evolutionary relationship, and expression pattern of PcG genes in *M. truncatula*, and investigate the potential role of these genes in important biological processes, we performed a genome-wide identification of PcG members and systematic analysis. The present study will provide valuable information for further research on the epigenetic regulation of forage.

## 2. Results

### 2.1. Identification of M. truncatula PcG Members

A total of 64 *M. truncatula* PcG genes were identified in the *M. truncatula* genome and generated the basic information (Table 1) on all PcG genes (including gene name, gene ID, location, open reading frame (ORF) length, number of exons, molecular weights (MW), isoelectric points (pI) value, and the grand average of hydropathicity (GRAVY). The results showed that the ORF lengths of the *M. truncatula* PcG genes ranged from 315 (*MtVRN1;23*, encodes 104 amino acids) to 3651 bp (*MtFIE;4*, encodes 1216 amino acids), the MW of the *M. truncatula* PcG proteins varied from 29.13 (MtRING1B;2) to 136.27 kDa (MtFIE;4), the pI value ranged from 3.97 (MtRING1B;2) to 9.99 (MtVRN1;24), and all of the *M. truncatula* PcG proteins were hydrophilic.

The secondary structure analysis (Table 2) of *M. truncatula* PcG proteins showed that the α-helixes ranged from 3.68% (MtFIE;16) to 50.79% (MtRING1B;2), the extended strand varied from 7.34% (MtRING1A) to 45.09% (MtFIE;16), the β-sheet extended from 1.71% (MtEMF1;1) to 14.42% (MtFIE;16), and the random coil ranged from 28.28% (MtFIE;18) to 73.20% (MtEMF1;1). Concurrently, the subcellular location analysis (Table 2) of *M. truncatula* PcG proteins indicated that most of the PcG proteins were located in the nucleus, whereas the prediction results of the two analysis programs were slightly different. For instance, Plant-mPLoc predicted that MtVRN1;12, MtVRN1;13, and MtVRN1;16 proteins were located in the nucleus, while they were predicted to be located in the chloroplast by WoLF PSORT. The MtFIE;11 protein was predicted to be located in the endoplasmic reticulum using the Plant-mPLoc method, while it was predicted to be located in the nucleus through the WoLF PSORT method. Moreover, we also built the tertiary structure (Figures S1 and S2) of the *M. truncatula* PcG proteins.

### 2.2. Gene Structure and Chromosome Location Analysis

**Figure 1:**
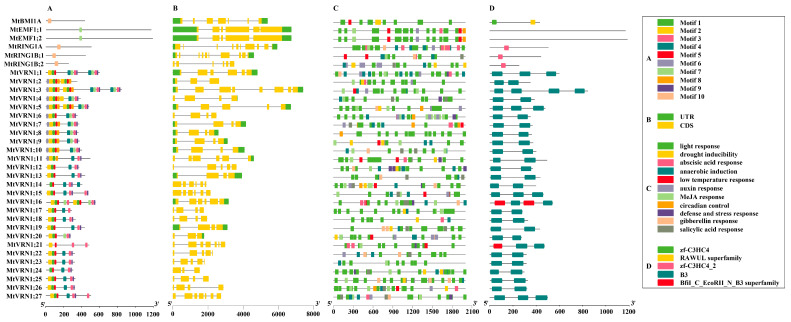
Motif composition (**A**), gene structure (**B**), cis-acting element prediction (**C**), and conserved domain analysis (**D**) of PRC1 complex members in *M. truncatula*.

The motif prediction of the PRC1 members showed (Figure 1A and Figure S3) that motif3 had the most occurrences (32 times) among all PRC1 members, while motif10 had the least occurrences (4 times). The *MtBMI1A* and RING1members possessed motif10, while motif7 was presented in all EMF members and several VRN1 members. It is noteworthy that *MtVRN1;3* contained the largest number of motifs (17 motifs), while there was only one motif in the RING1, BMI1, and EMF1 members. The exon–intron structure of PRC1 members (Figure 1B) indicated that the number of exons of all members ranged from 3 to 10, and that the majority of PRC1 members contained four exons (12 genes). The EMF1 members exhibited a similar exon–intron structure, while the exon–intron structure of the VRN1 members was quite different. For example, *MtVRN1;2* and *MtVRN1;3* contained three and eight exons, respectively. The cis-acting elements in the promoter of the PRC1 members (Figure 1C) were analyzed and the results showed that the light-responsive elements were detected in 33 PRC1 genes. However, only eight genes contained the circadian control elements, which were mainly enriched in the EMF1 (two genes) and VRN1 (six genes) members. The abscisic acid (ABA) response and methyl jasmonate (MeJA) responsive elements also had more distributions in the PRC1 members (24 and 25 genes, respectively), but fewer than the light response and anaerobic induction (27 genes) elements. Additionally, the statistical results (Figure S4A) of cis-acting elements suggested that there were two types of elements involved in the growth and development of the PRC1 members, which included the light response and circadian rhythm responsive elements. Five types of cis-acting elements were related to plant hormones, including ABA, indole-3-acetic acid (IAA), MeJA, gibberellin (GA), and salicylic acid (SA) elements. Additionally, four types of cis-acting elements were involved in the environmental stimuli, including the drought responsive, anaerobic response, low temperature response, and defense/stress responsive elements. The conserved domain analysis of the PRC1 members (Figure 1D) revealed that the type of domains varied between different components. Among them, the BMI1 component contained the zf-C3HC4 and RAWUL-superfamily functional domains, the RING1 component possessed only the zf-C3HC4_2 domain, and the VRN1 component contained the B3 and Bfil_C_EcoRll_N_B3 superfamily functional domains. There was a similar type of domain among members of the VRN1 component that contained at least two B3 domains, of which *MtVRN1;3* had the greatest number of B3 domains (four domains).

**Figure 2:**
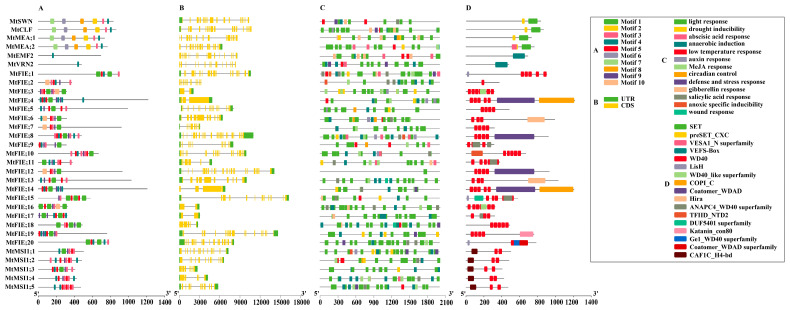
Motif composition (**A**), gene structure (**B**), cis-acting element prediction (**C**), and conserved domain analysis (**D**) of PRC2 complex members in *M. truncatula*.

The motif prediction of the PRC2 complex members showed (Figure 2A and Figure S5) that motif3 had the most occurrences (40 times) among PRC1 members, whereas motif2, motif6, motif7, and motif8 had the least occurrences (four times). It is noted that the *MtFIE;4* contained the largest number of motifs (10 motifs), while there was only one motif in the *EMF2* and *VRN2* members. *MtSWN*, *MtCLF*, *MtMEA;1*, and *MtMEA;2* had similar motif compositions, and this similar motif composition was also displayed between members in the FIE and MSI1 components. The exon–intron structure of the PRC2 members (Figure 2B) showed that the number of exons of all members varied from 2 to 24. The members of the SWN/CLF/MEA and FIE components exhibited different exon–intron structures. For instance, *MtMtFIE;3*, *MtFIE;16*, and *MtFIE;17* contained the least number of exons (two exons), while *MtMtFIE;8* and *MtMtFIE;12* possessed the largest number of exons (23 exons). The cis-acting elements in the promoter of the PRC2 members (Figure 2C) were analyzed and the results showed that the light-responsive elements were detected in 31 PRC2 genes, but only two genes contained the wound-responsive elements. The abscisic acid response and MeJA responsive elements also had higher distributions in the PRC2 members (20 and 19 genes, respectively), but fewer than the light response and anaerobic induction (28 genes) elements. Furthermore, the statistical results (Figure S4B) of the cis-acting elements implied that the elements related to growth, development, and plant hormones in the PRC2 members were consistent with the PRC1 members. The elements related to the environmental stimuli of PRC2 members not only included the four elements consistent with PRC1 members but also contained anoxic specific inducibility response and wound-responsive elements. The conserved domain analysis of the PRC2 members (Figure 2D) revealed that the type of domains was more diverse than the PRC1 members, and that the type of domains of members within each component was similar. Among them, the SWN/CLF/MEA component contained SET and preSET_CXC functional domains, while the EMF2/VRN2 component only possessed the VEFS-Box domain. Interestingly, though the FIE component contained 12 functional domains, WD40 was the functional domain shared by each member. The members of the MSI1 component both contained CAF1C_H4-bd and WD40 functional domains, and the type and number of domains between members were consistent.

**Figure 3:**
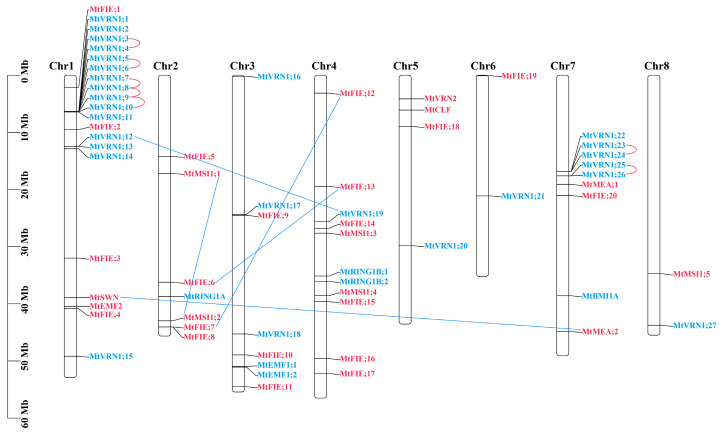
Chromosome distribution and gene duplication of PcG members in *M. truncatula*, the red and blue fonts indicate members of the PRC1 and PRC2, respectively. The red and blue lines represent clustered tandem duplication and segmental duplication, respectively.

The results of the chromosome location of *M. truncatula* PcG members showed that all members were distributed unevenly over eight chromosomes (Figure 3). Chromosome 1 contained the highest number of PcG members (21 genes), while chromosomes 6 and 8 had the lowest numbers of PcG members (two genes). Simultaneously, eleven and nine PcG genes were, respectively, located on chromosomes 4 and 7. There was no correlation between the length of the chromosome and the number of PcG members it contained. Additionally, the highest number of PRC1 members were located on chromosome 1 (15 genes), whereas only one PRC1 member was located on chromosomes 2, 5, 6, and 8. Most of the PRC2 members were distributed on chromosome 4 (eight genes), while only one PRC2 member was distributed on chromosomes 6 and 8. The FIE component (except chromosome 8) and VRN1 component (except chromosome 2) were located on all chromosomes.

We further analyzed the gene duplication events of *M. truncatula* PcG members. The results showed that there were 7173 (14.22%) tandem replicated genes and 6642 (13.17%) segmental replicated genes (Table S1-1) through the whole-genome analysis of *M. truncatula*. Among the PcG members of *M. truncatula*, 14 (21.88%) and 12 (18.75%) PcG genes were, respectively, identified as tandem duplication and segmental duplication (Table S1-2). Concurrently, we found that eight PcG genes (*MtVRN1;3*/*4*, *MtVRN1;5*/*6*, *MtVRN1;7*/*8*, *MtVRN1;8*/*9*, *MtVRN1;9*/*10*) were clustered into five tandem duplication event regions on chromosome 1, while four PcG genes (*MtVRN1;23*/*24*, *MtVRN1;25*/*26*) were clustered into two tandem duplication event regions on chromosome 7 (Figure 3). In addition, we identified five segmental duplication events, including 10 PcG genes (*MtVRN1;12*/*19*, *MtSWN*/*MtMEA;2*, *MtFIE;6*/*13*, *MtFIE;7*/*12*, *MtMSI1;1*/*2*).

### 2.3. Protein–Protein Interaction Analysis

**Figure 4:**
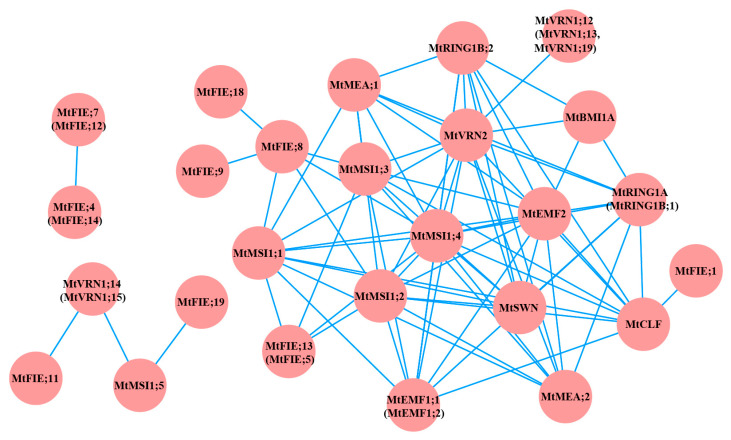
Prediction of protein–protein interaction among PcG members in *M. truncatula*.

To further understand the interaction between the PcG proteins of *M. truncatula*, we constructed the protein interaction networks of 64 PcG proteins using the online analysis website STRING (Figure 4, Attached Table S2). The results indicated that 34 PcG proteins interacted with each other, generating 77 protein–protein interactions. Among them, MtMSI1;4 and MtVRN2 had the largest number of protein–protein interactions (13 interactions), while MtFIE;1, MtFIE;4 (MtFIE;14), MtFIE;7 (MtFIE;12), MtFIE;9, MtFIE;11, MtFIE;18, MtFIE;19, and MtVRN1;12 (MtVRN1;13, MtVRN1;19) had the lowest number of protein–protein interactions (one interaction). In the protein interaction networks of *A. thaliana* PcG proteins, we found that AtLHP1, AtFIE, AtRING1A, and AtRING1B possessed the largest number of interacting proteins (Figure S6).

### 2.4. Phylogenetic Analysis and Synteny Analysis

We performed a multiple protein sequence alignment involving 64 *M. truncatula* PcG members and 16 *A. thaliana* PcG members to further understand the structure and characteristics of the *M. truncatula* PcG members (from Figure S7-1 to Figure S7-8). The results showed that 1 PcG protein contained both conserved zf-C3HC4 and RAWUL domains, 3 PcG proteins contained a conserved zf-C3HC4_2 domain, 27 PcG proteins contained at least 2 conserved B3 domains, 4 PcG proteins contained both conserved preSET_CXC and SET domains, 2 PcG proteins contained a conserved VEFS-Box domain, 20 PcG proteins contained a WD40 domain, and 5 PcG proteins contained both conserved CAF1C_H4-bd and WD40 domains.

**Figure 5:**
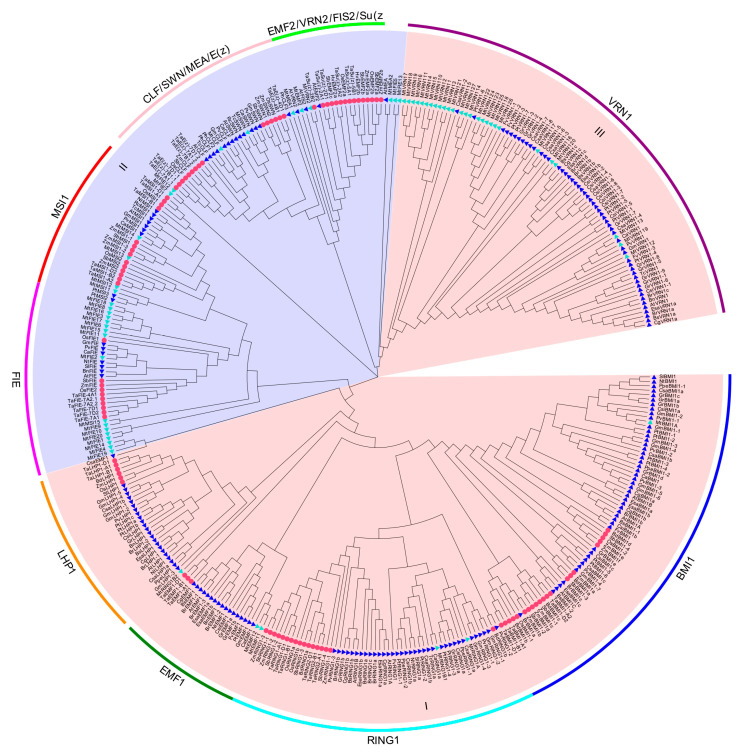
The rootless phylogenetic tree of PcG proteins in *M. truncatula* and other species. Different colored arcs indicate different components of PRC1 and PRC2. The turquoise triangle, the red circle and the blue triangle represent the PcG members of *M. truncatula*, monocots and dicots, respectively. At: *Arabidopsis thaliana*, Bd: *Brachypodium distachyon*, Bn: *Brassica napus*, Br: *Brassica rapa*, Bs: *Boechera stricta*, Ca: *Cicer arietinum*, Cg: *Capsella grandiflora*, Csa: *Cucumis sativus*, Csi: *Citrus sinensis*, Esa: *Eutrema salsugineum*, Fv: *Fragaria vesca*, Gm: *Glycine max*, Gr: *Gossypium raimondii*, Hv: *Hordeum vulgare*, Mt: *Medicago truncatula*, Nt: *Nicotiana tomentosiformis*, Os: *Oryza sativa*,Ph: *Petunia hybrida*, Ppe: *Prunus persica*, Pt: *Populus trichocarpa*, Pv: *Phaseolus vulgaris*, Rc: *Ricinus communis*, Sb: *Sorghum bicolor*, Sl: *Solanum lycopersicum*, Ta: *Triticum aestivum*, Tc: *Theobroma cacao*, Zm: *Zea mays*.

Moreover, in order to obtain a phylogenetic insight into the relationship of PcG proteins in *M. truncatula*, a phylogenetic tree was constructed based on the sequence alignment, involving 375 PcG proteins from 27 species (Figure 5). The results of the phylogenetic analysis indicated that all PcG proteins were divided into three large groups. Group I and group III belonged to the PRC1 complex, while group II belonged to the PRC2 complex. Group I included four subgroups, which contained BMI1, RING1, EMF1, and LHP1; group II contained four subgroups which were composed of FIE, MSI1, CLF/MEA/SWN/E(z), and EMF2/VRN2/FIS2/Su(z); and group III had only one subgroup which consisted of VRN1. The VRN1 component possessed the largest number of members (80 members), whereas the EMF2/VRN2/FIS2/Su(z) component had the least number of members (20 members). It is noteworthy that we did not find the members of the LHP1 and FIS2 components in the *M. truncatula* genome database (v4.0). The phylogenetic tree displayed that PcG proteins in several components could be divided into different clades according to monocot and dicot plants, which included BMI1, EMF1, MSI, CLF/MEA/SWN/E(z), FIE, and LHP1. In RING1 and EMF2/VRN2/FIS2/Su(z), nevertheless, the PcG proteins of monocot and dicot plants were grouped in the same clade. In addition, we also found that *M. truncatula* MtBMI1A protein and *G. max* GmBMI1-1 protein belonged to the same clade. The VRN1;13, MtRING1B1, MtEMF1;1, MtEMF1;2, MtFIE2, MtMSI1;4, and MtSWN proteins of *M. truncatula* were grouped in the same clade with the corresponding proteins in *C. arietinum*. The *M. truncatula* MtMEA2 was located in the same clade with *A. thaliana* AtMEA, while the *M. truncatula* MtVRN2 protein was clustered into the same clade with *T. aestivum* TaSu(z)-2B2.

**Figure 6:**
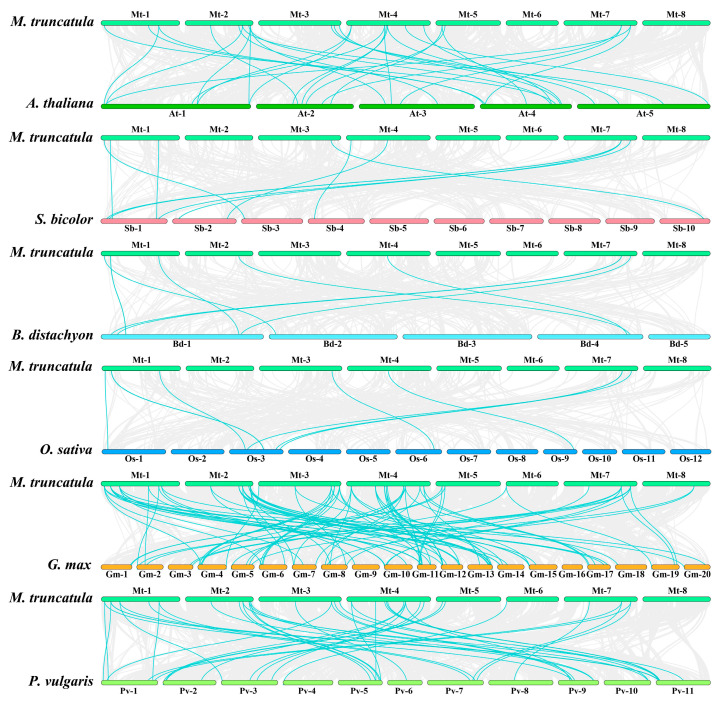
Synteny analysis of PcG gene between *M. truncatula* and six representative plants. The gray line represents the syntenic block in plant genomes, and the turquoise line represents the PcG collinear gene pair.

In order to further understand the syntenic relationships between *M. truncatula* and other species, we constructed six comparative syntenic diagrams between *M. truncatula* and the representative species, including three dicot plants (*A. thaliana*, *G. max*, and *P. vulgaris*) and three monocot plants (*B. distachyon*, *O. sativa*, and *S. bicolor*) (Figure 6 and Table S3). Among them, *M. truncatula* PcG genes had the most collinearity relationships with *G. max* (36 genes), followed by *P. vulgaris* (31 genes) and *A. thaliana* (21 genes), while there were few collinearity relationships with the *O. sativa*, *B. distachyon*, and *S. bicolor* (8 genes). The number of orthologous pairs between *M. truncatula* and *G. max*, *P. vulgaris*, *A. thaliana*, *O. sativa*, *B. distachyon*, and *S. bicolor* was 87, 43, 31, 9, 9, and 10, respectively. Meanwhile, we found that nine genes (*MtBMI1A*, *MtRING1A*, *MtVRN1;12*, *MtMEA;2*, *MtFIE;1*, *MtFIE;7*, *MtFIE;11*, *MtFIE;12*, *MtFIE;15*) possessed four syntenic gene pairs. There were five common syntenic genes in all species (*MtBMI1A*, *MtSWN*, *MtMEA;2*, *MtFIE;1*, and *MtMSI1;3*). The present analysis also identified some syntenic blocks containing PcG genes (Table S4). The 87 orthologous gene pairs composed of 36 PcG genes of *M. truncatula* and 76 genes of *G. max* were distributed in 70 syntenic blocks, and 44 orthologous gene pairs composed of 31 PcG genes of *M. truncatula* and 35 genes of *P. vulgaris* were distributed in 35 syntenic blocks.

### 2.5. Expression Pattern Analysis

#### 2.5.1. Microarray Expression Data and Transcriptome Sequencing Data

**Figure 7:**
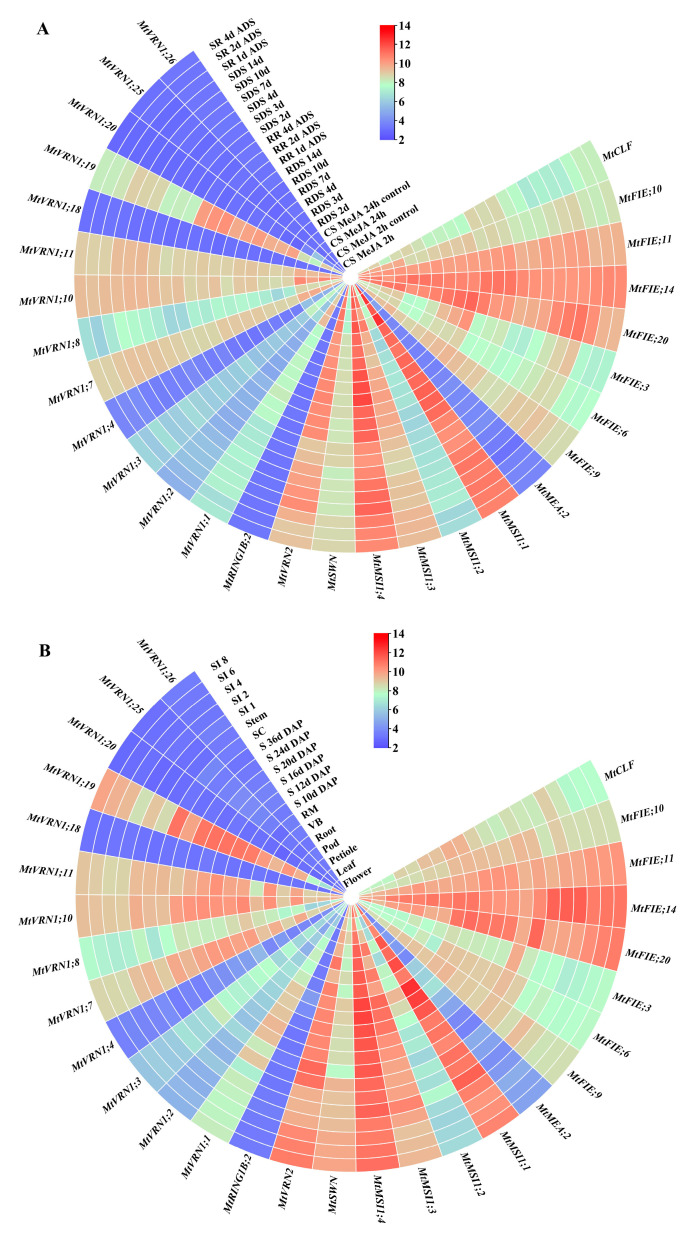
The expression patterns of PcG members in different treatments (**A**) and different tissues/developmental stages (**B**) were analyzed based on microarray expression data. CS, cell suspension; RDS, root drought stress; RR, root rehydration; SDS, shoot drought stress; SR, shoot rehydration; VB, vegetative Buds; RM, root meristem; S, seed; DAP, days after pollination; SC, seed coat; SI, stem internode. Scale bar represents the log2 normalized expression. Red color represents higher expression while blue represents lower expression.

The microarray expression data showed that different treatment conditions could regulate the expression levels of *M. truncatula* PcG genes (Figure 7A). The MeJA treatment expression analysis illustrated that four PcG genes (*MtMSI1;3*, *MtFIE;14*, *MtVRN1;10*, and *MtVRN1;19*) were down-regulated. Under drought stress, the expression levels of five PcG genes (*MtMSI1;2*, *MtMSI1;4*, *MtFIE;3*, *MtFIE;6* and *MtFIE;20*) increased with the treatment time. However, the expression of these genes was down-regulated after rehydration. The expression level of *MtFIE;14* decreased under drought stress but increased after rehydration. Simultaneously, the microarray data also suggested that the *M. truncatula* PcG genes had potential functions in different tissues and seed development processes (Figure 7B). *MtMSI1;1* was highly expressed in vegetative buds, pods, seed coats, and stems, while *MtMSI1;4* had higher expression levels in pods, roots, vegetative buds, the root meristem, seed coats, and stems. The expression levels of these two genes fluctuated during seed development, whereas they had decreased expression with an increase in the number of stem internodes. In addition, *MtVRN1;19*, *MtVRN2*, and *MtFIE;20* had high expression levels in seed coats and *MtFIE;14* possessed a high expression level in stems.

**Figure 8:**
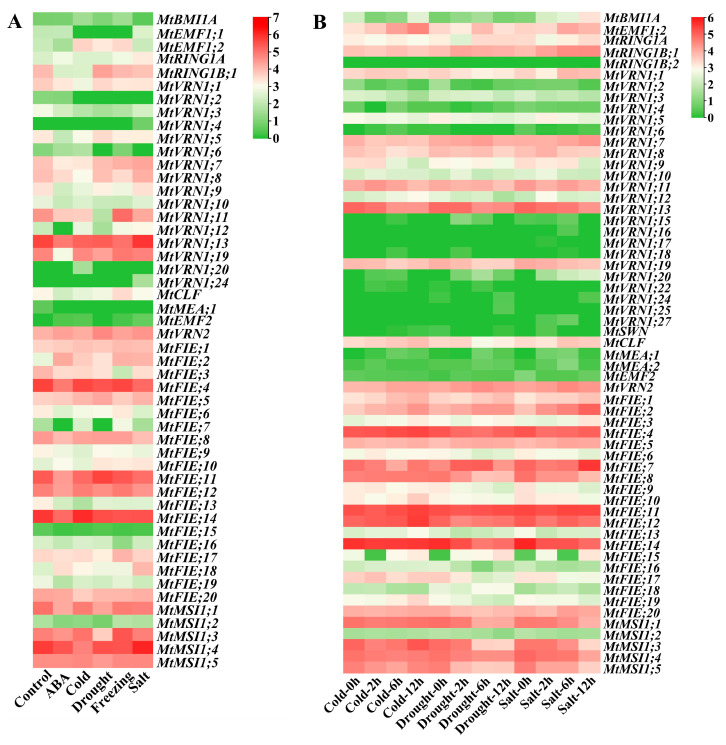
The expression patterns of PcG genes under different treatment conditions were analyzed based on RNA-seq data. (**A**) RNA-seq data from ABA, cold, drought, freezing and salt treatments. (**B**) RNA-seq data from cold, drought, and salt treatments at different times. Scale bar represents the log2 normalized expression. Red color represents higher expression while green represents lower expression.

**Figure 9:**
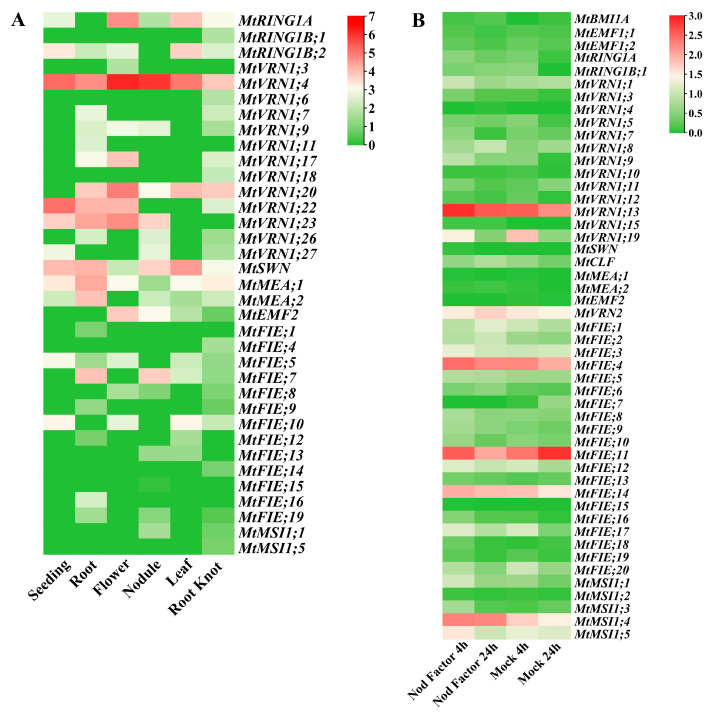
The expression patterns of PcG genes in different tissue (**A**) and different treatment of root (**B**) were analyzed based on RNA-seq data. Scale bar represents the log2 normalized expression. Red color represents higher expression while green represents lower expression.

We obtained the RNA-seq data of *M. truncatula* through the NCBI database and analyzed the expression patterns of PcG genes under different hormones, different stress conditions (Figure 8), different tissues (Figure 9A), and different treatments of root (Figure 9B). Regarding cold stress, the expression levels of *MtRING1B;1*, *MtVRN1;1*, *MtVRN1;8*, *MtVRN1;9*, *MtVRN1;11*, *MtVRN1;13*, *MtFIE;11*, *MtFIE;13*, and *MtMSI1;4* were significantly down-regulated, while the expression levels of *MtEMF1;2*, *MtVRN1;20*, *MtFIE;2*, and *MtFIE;7* were significantly up-regulated (Figure 8A). Under cold stress, the expression levels of *MtFIE;17*, *MtVRN1;1*, *MtVRN1;9*, and *MtVRN1;11* first increased and then decreased with the treatment time, while the expression of *MtFIE;7*, *MtFIE;10*, *MtFIE;15*, *MtBMI1A*, *MtMSI1;3*, and *MtMSI1;5* exhibited an opposite trend. In addition, the expression levels of *MtEMF1;2*, *MtVRN1;12*, *MtCLF*, *MtFIE;1*, *MtFIE;2*, and *MtFIE;19* increased with the treatment time, whereas the expression of *MtVRN1;3*, *MtVRN1;13*, and *MtFIE;18* had an opposite trend (Figure 8B). For drought stress, the expression levels of 14 PcG genes (*MtEMF1;1*, *MtVRN1;2*, *MtVRN1;3*, *MtVRN1;6*, *MtVRN1;11*, *MtVRN1;13*, *MtVRN1;19*, *MtFIE;3*, *MtFIE;7*, *MtFIE;14*, *MtFIE;17*, *MtMSI1;1*, *MtMSI;2*, and *MtMSI1;3*) were down-regulated, while the expression levels of eight PcG genes (*MtEMF1;2*, *MtRING1B;1*, *MtVRN1;5*, *MtVRN2*, *MtFIE;2*, *MtFIE;5*, *MtFIE;10*, and *MtFIE;11*) were up-regulated (Figure 8A). Under drought stress, the expression levels of *MtBMI1A*, *MtRING1B;1*, *MtVRN1;7*, *MtVRN1;15*, *MtVRN1;20*, *MtVRN2*, and *MtFIE;7* first increased and then decreased with the treatment time, while the expression of *MtFIE;1* and *MtFIE;13* displayed an opposite trend. The expression levels of *MtFIE;9*, *MtFIE;19*, and *MtMSI1;2* decreased with the treatment time (Figure 8B). For salt stress, the expression levels of 13 PcG genes (*MtBMI1A*, *MtRING1B;1*, *MtVRN1;1*, *MtVRN1;2*, *MtVRN1;6*, *MtVRN1;11*, *MtFIE;3*, *MtFIE;4*, *MtFIE;6*, *MtFIE;8*, *MtFIE;11*, *MtFIE;12*, and *MtFIE;19*) were down-regulated, whereas the expression levels of 10 PcG genes (*MtRING1A*, *MtVRN1;4*, *MtVRN1;7*, *MtVRN1;8*, *MtVRN1;12*, *MtVRN1;24*, *MtFIE;2*, *MtFIE;5*, *MtFIE;18*, and *MtVRN2*) were up-regulated (Figure 8A). Under salt stress, the expression levels of eight PcG genes (*MtFIE;8*, *MtFIE;14*, *MtVRN1;13*, *MtVRN1;19*, *MtMSI1;1*, *MtMSI1;3*, *MtMSI1;4*, and *MtMSI1;5*) decreased with treatment time, while five PcG genes (*MtBMI1A*, *MtRING1B;1*, *MtVRN1;7*, *MtVRN1;20*, and *MtFIE;2*) showed an opposite trend. The expression levels of five PcG genes (*MtRING1A*, *MtCLF*, *MtFIE;4*, *MtFIE;7*, and *MtFIE;13*) first decreased and then increased with the treatment time (Figure 8B). In ABA treatment, the expression levels of 19 PcG genes (*MtEMF1;2*, *MtRING1B;1*, *MtVRN1;5*, *MtVRN1;7*, *MtVRN1;9*, *MtVRN1;11*, *MtVRN1;12*, *MtVRN1;13*, *MtVRN1;19*, *MtFIE;3*, *MtFIE;4*, *MtFIE;6*, *MtFIE;7*, *MtFIE;8*, *MtFIE;11*, *MtFIE;13*, *MtFIE;14*, *MtFIE;19*, and *MtMSI1;1*) were down-regulated, whereas the expression levels of five PcG genes (*MtRING1A*, *MtVRN2*, *MtFIE;2*, *MtFIE;10*, and *MtFIE;18*) were up-regulated (Figure 8A). Regarding freezing stress, the expression levels of eight PcG genes (*MtEMF1;1*, *MtVRN1;1*, *MtVRN1;2*, *MtVRN1;8*, *MtVRN1;9*, *MtVRN1;13*, *MtFIE;3*, and *MtFIE;16*) were down-regulated, while those of nine PcG genes (*MtBMI1A*, *MtEMF1;2*, *MtVRN1;11*, *MtVRN1;12*, *MtCLF*, *MtFIE;2*, *MtFIE;7*, *MtFIE;17*, and *MtMSI1;3*) were up-regulated (Figure 8A).

The expression data of *M. truncatula* PcG genes in different tissues (Figure 9A) illustrated that *MtVRN1;4*, *MtVRN1;22*, *MtVRN1;23*, and *MtSWN* were highly expressed in seeding, while *MtVRN1;4*, *MtVRN1;20*, *MtVRN1;22*, *MtVRN1;23*, *MtSWN*, *MtMEA;1*, *MtMEA;2*, and *MtFIE;7* also had high expression levels in the roots. Seven PcG genes (*MtRING1A*, *MtVRN1;4*, *MtVRN1;17*, *MtVRN1;20*, *MtVRN1;22*, *MtVRN1;23*, and *MtEMF2*) possessed high expression levels in flower; four PcG genes (*MtVRN1;4*, *MtVRN1;23*, *MtSWN*, *MtFIE;7*) had high expression levels in nodules; five PcG genes (*MtRING1A*, *MtRING1B;2*, *MtVRN1;4*, *MtVRN1;20*, and *MtSWN*) were highly expressed in leaves; and *MtVRN1;4*, *MtVRN1;20*, and *MtMEA;1* had high expression levels in root knots. For the different treatments of roots (Figure 9B), we found that the expression levels of *MtVRN1;13*, *MtVRN1;19*, *MtFIE;4*, *MtFIE;14*, *MtFIE;17*, *MtMSI1;4*, and *MtMSI1;5* decreased with the treatment time. The expression levels of *MtCLF* and *MtVRN2* increased with treatment under Nod factor treatment, but displayed an opposite trend under mock treatment. The expression levels of *MtFIE;11* decreased with treatment under Nod factor treatment, while they exhibited an opposite trend under mock treatment.

#### 2.5.2. Co-Expression and GO Enrichment Analysis of PcG Genes

**Figure 10:**
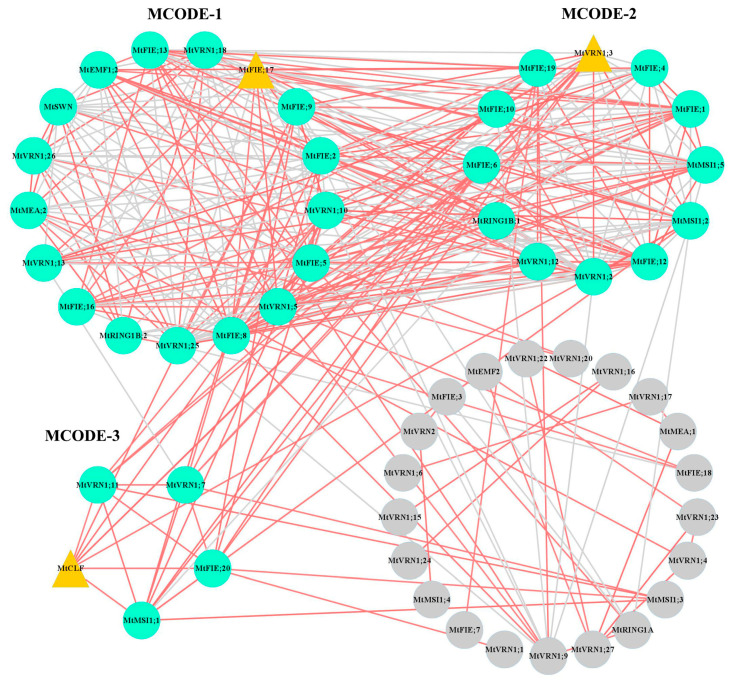
The co-expression network of *M. truncatula* PcG genes. Green circle and yellow triangle separately indicate the members and hub genes of three clusters in MCODE. Gray circle indicates the unclustered PcG genes. Red lines and gray lines represent the positive correlation value and negative correlation value, respectively.

We used downloaded microarray expression data (Figure 7) and RNA-seq data (Figure 8 and Figure 9) to construct a co-expression network of *M. truncatula* PcG genes. The results showed that three clusters (MCODE-1, MCODE-2, and MCODE-3) with high clustering scores were generated by MCODE; and *MtFIE;17*, *MtVRN1;3*, and *MtCLF* were the hub genes in three clusters (Figure 10). From the analysis of the GO enrichment of the top two MCODEs (MCODE-1 and MCODE-2), we found that the genes of the same cluster were co-regulated in specific biological processes (Table S5). Taking MCODE-1 with the most GO enriched terms as an example, MCODE-1 possessed 10 GO terms related to the regulation of metabolic process (GO:0080090, GO:0019222, GO:0031323, GO:0060255, GO:0019219, GO:0051171, GO:0051252, GO:0016070, GO:0006139, GO:0006807), 9 terms related to the regulation of biosynthetic process (GO:0031326, GO:0032774, GO:0009889, GO:0010556, GO:0034645, GO:0009059, GO:0050789, GO:0044249, GO:0009058), and 6 terms related to the regulation of transcription and gene expression (GO:0045449, GO:0010468, GO:0006355, GO:0006350, GO:0006351, GO:0010467).

#### 2.5.3. The Expression Patterns of PcG Genes in qRT-PCR Analysis

**Figure 11:**
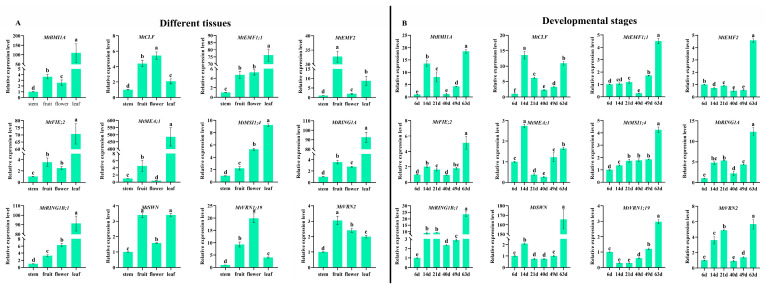
The relative expression levels of PcG genes in different tissues (**A**) and different development stages (**B**). Different letters represent significant differences (*p* < 0.05) using ANOVA and Duncan test among samples. Error bars indicate standard deviation.

In this study, we screened 12 representative *M. truncatula* PcG genes for qRT-PCR analysis (Figure 11). We set the stem as the control sample in different tissues; the expression data (Figure 11A) showed that most genes had higher expression levels in leaves than in other tissues, while four PcG genes (*MtCLF*, *MtVRN2*, *MtVRN1;19*, and *MtEMF2*) had higher expression levels in fruits/flowers than in leaves. Concurrently, the expression patterns of six PcG genes (*MtBMI1A*, *MtEMF2*, *MtFIE;2*, *MtMEA;1*, *MtRING1A*, and *MtSWN*) were similar, showing high relative expression levels in fruits and leaves. *MtEMF1;1*, *MtMSI1;4*, and *MtRING1B;1* showed similar expression patterns and had the highest expression in leaves. *MtCLF* and *MtVRN1;19* had similar expression patterns and possessed high relative expression levels in fruits and flowers. We set the 6th day after germination as the control sample; the expression data (Figure 11B) suggested that two PcG genes (*MtCLF* and *MtMEA;1*) had the highest relative expression levels at the 14th day after germination, while the remaining 10 PcG genes had the highest relative expression levels at the 63rd day after germination. Furthermore, six PcG genes (*MtBMI1A*, *MtCLF*, *MtMEA;1*, *MtRING1A*, *MtRING1B;1*, and *MtVRN2*) had similar expression patterns, showing fluctuated expression patterns during the plant development. The expression patterns of the other six PcG genes (*MtEMF1;1*, *MtEMF2*, *MtFIE;2*, *MtMSI1;4*, *MtSWN*, and *MtVRN1;19*) were similar; they had low relative expression levels from the 6th to the 49th day after germination and these significantly increased at the 63rd day after germination.

**Figure 12:**
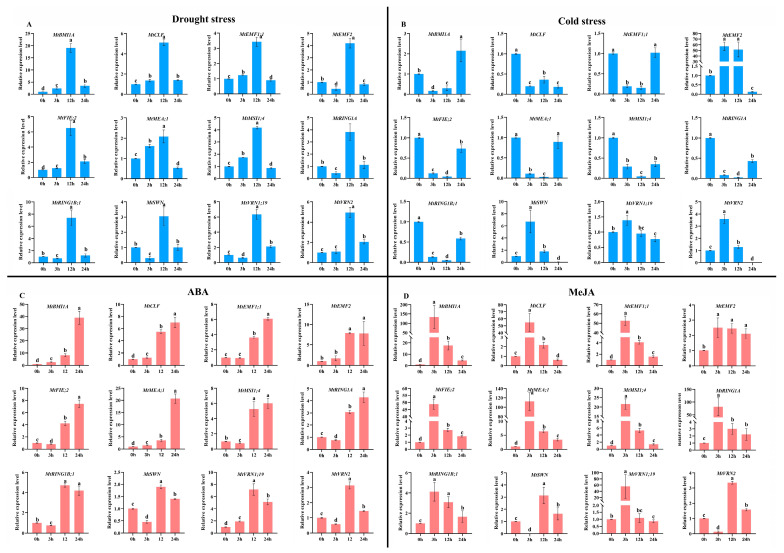
The relative expression levels of PcG genes under drought stress (**A**), cold stress (**B**), abscisic acid (**C**), and methyl jasmonate (**D**). Different letters represent significant differences (*p* < 0.05) using ANOVA and Duncan test among samples. Error bars indicate standard deviation.

Meanwhile, we set the 0 h after treatment as the control sample and analyzed the expression patterns of 12 PcG genes under different stress and hormone treatments (Figure 12). Under drought stress (Figure 12A), the relative expression levels of all PcG genes were significantly up-regulated (*p* < 0.05) 12 h after treatment, while the relative expression levels of all PcG genes were significantly down-regulated (*p* < 0.05) 24 h after treatment. Additionally, seven PcG genes (*MtBMI1A*, *MtCLF*, *MtEMF1;1*, *MtFIE;2*, *MtMEA;1*, *MtMSI1;4*, and *MtVRN2*) had similar expression patterns, which first increased and then decreased with the treatment time. Another five PcG genes (*MtEMF2*, *MtRING1A*, *MtRING1B;1*, *MtSWN*, and *MtVRN1;19*) had similar expression patterns and showed fluctuated expression patterns during the drought stress. For cold stress (Figure 12B), seven PcG genes (*MtBMI1A*, *MtEMF1;1*, *MtFIE;2*, *MtMEA;1*, *MtMSI1;4*, *MtRING1A*, and *MtRING1B;1*) exhibited similar expression patterns, which decreased firstly and then increased with the treatment time. Four PcG genes (*MtEMF2*, *MtSWN*, *MtVRN1;19*, and *MtVRN2*) showed similar expression patterns, which first increased and then decreased with the treatment time. The relative expression level of the *MtCLF* gene showed a fluctuating trend. In the ABA treatment (Figure 12C), five PcG genes (*MtBMI1A*, *MtCLF*, *MtEMF1;1*, *MtEMF2*, and *MtMEA;1*) had similar expression patterns, which continually increased with the treatment time. The relative expression level of *MtVRN1;19* first increased and then decreased with the treatment time. Three PcG genes (*MtFIE;2*, *MtMSI1;4*, and *MtRING1A*) had similar expression patterns, which first decreased and then increased with the treatment time. Three PcG genes (*MtRING1B;1*, *MtSWN*, and *MtVRN2*) had similar expression patterns, which showed fluctuated expression patterns during the ABA treatment. As for the MeJA treatment (Figure 12D), 10 PcG genes (*MtBMI1A*, *MtCLF*, *MtEMF1;1*, *MtEMF2*, *MtFIE;2*, *MtMEA;1*, *MtMSI1;4*, *MtRING1A*, *MtRING1B;1*, and *MtVRN1;19*) had similar expression patterns, which first increased and then decreased with the treatment time. Another two PcG genes (*MtSWN* and *MtVRN2*) had similar expression patterns, which exhibited fluctuated expression patterns during the MeJA treatment.

### 2.6. Identification of Differentially Expressed PcG Genes Based on RNA-seq Data

**Figure 13:**
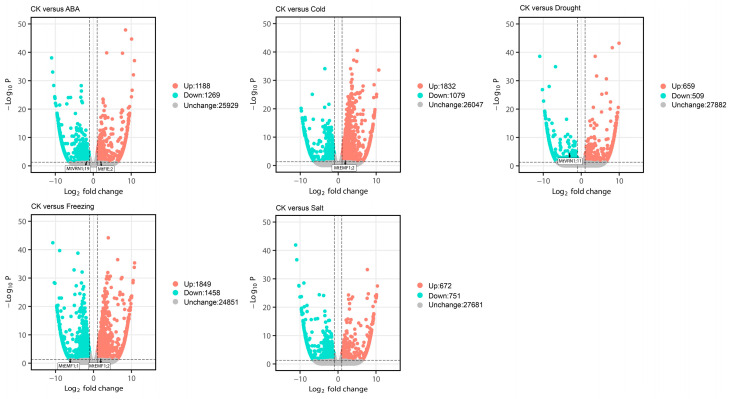
Identification of the differentially expressed PcG genes under different stress.

**Figure 14:**
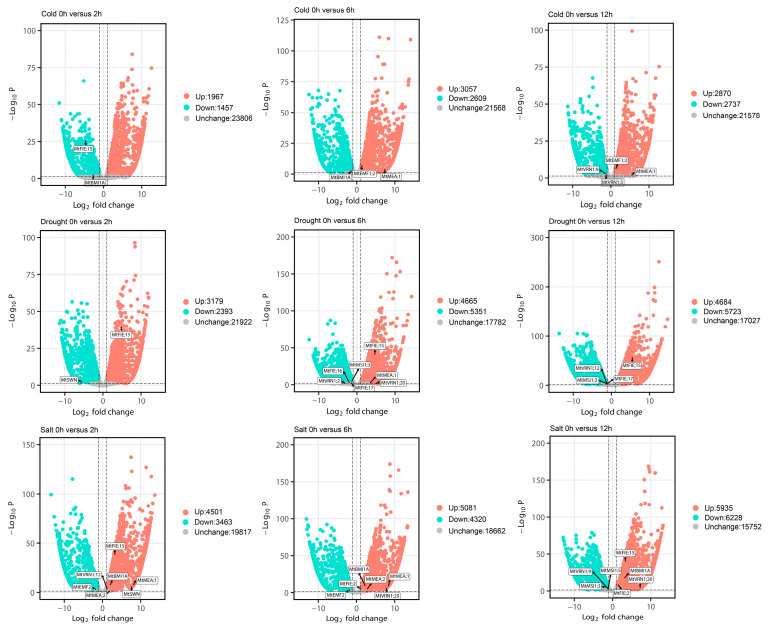
Identification of the differentially expressed PcG genes in different stress process.

We identified several PcG genes in *M. truncatula* that were differentially expressed under different hormones and stress treatments. Two differentially expressed PcG genes were identified in ABA and freezing treatments, and one differentially expressed PcG gene was identified under cold and drought stresses (Table S6, Figure 13). Two, three, and four differentially expressed PcG genes were identified in three DEG sets under the cold stress process. Two, seven, and four differentially expressed PcG genes were identified in the three DEG sets under the drought stress process. Seven, six, and seven differentially expressed PcG genes were identified in the three DEG sets under the salt stress process (Table S6, Figure 14). However, we found no differentially expressed PcG genes in different tissue sites (Figure S8).

## 3. Discussion

### 3.1. Identification of M. truncatula PcG Members and Structure Analysis

We obtained 16 PcG members of *A. thaliana* following the statistics of previous studies. The protein sequences of these members had an average molecular weight of 65.14 kDa and an average length of 579 amino acids [7,25,61]. In the present study, 64 PcG members were identified in *M. truncatula* based on the sequence similarity and conserved domain. The protein sequences of 64 PcG members had an average molecular weight of 61.37 kDa, with an average of 543 amino acids. Hence, this result of the study was similar to that reported in *A. thaliana*. We found 64 PcG members in the *M. truncatula* genome, which was higher than the number found in previous research [57,58,59,60]. Several researchers identified 10 PcG proteins in *M. truncatula* [57,58] and 48 PcG proteins in *T. aestivum* [62]. Therefore, it is more likely that the number of PcG members in different species was associated with the genome size of the species, ploidy level, analytical method used, and other factors. It is noted that the previously reported *MtLHP1* gene was identified based on another genome database (Mt3.5v5 version) [60], whereas the *LHP1* gene of *M. truncatula* was not identified in this study. Moreover, it was previously reported that the members of FIS2 and MEA have not been identified in *O. sativa*, *T. aestivum*, *Z. mays*, and *M. truncatula* [57,58,62,63]. Similarly, we did not identify the member of the FIS2 component in the present study. In contrast, two *MEA* genes of *M. truncatula* (*MtMEA;1* and *MtMEA;2*) were identified in this study, which was consistent with the report of Che [59].

We found that two EMF1 proteins (*MtEMF1;1* and *MtEMF1;2*) in *M. truncatula* did not have annotated conserved domains, but they had common motifs with other members of PRC1, which was consistent with previous reports [20,23]. A previous report suggested that the number of B3 domains in 27 VRN1 homologous proteins ranged from two to four. Among them, the VRN1 protein in hexaploid bread wheat contained four B3 conserved domains, and only two B3 domains were found in the VRN1 protein of *A. thaliana* [62]. The types of domains conserved among PRC1 members in *M. truncatula* were quite different, suggesting that these members had independent functions. Nevertheless, MtSWN, MtCLF, and MtMEAs proteins contained SET and preSET_CXC domains, and MtFIEs and MtMSI1 proteins had a common WD40 domain, revealing that the PRC2 members in *M. truncatula* had partial functional redundancy. This result was similar to that reported in *A. thaliana* [64].

Subcellular localization analysis could contribute to a better understanding of the biological function of the target gene [65]. In the present study, we predicted that the MtBMI1A, MtEMF1s, MtRING1A/B, MtCLF, MtSWN, MtVRN2, and MtEMF2 proteins were located in the nucleus, which was consistent with our observation in the AtBMI1, AtEMF1, AtRING1A/B, AtCLF, and AtVRN2 proteins of *A. thaliana* [5,23,36,66,67]; the VvRING1a/b protein of *V. vinifera* [68]; the ZmCLF and ZmSWN proteins of *Z. mays* [63]; and the OsEMF2b protein of *O. sativa* [69]. In addition, we found that the MtVRN1 protein may be located in multiple locations, such as the nucleus, chloroplast, cell wall, and cytoplasm, while the VRN1 homologous protein in *G. max* was only localized to the nucleus [70]. MtFIE protein also predicted that this protein may be located in multiple locations, including the nucleus, cytoplasm, chloroplast, endoplasmic reticulum, Golgi apparatus, and chloroplast, which was similar to the findings reported in *A. thaliana* [71]. We predicted that the MtMSI1 protein may be located in the nucleus, cytoplasm, and mitochondria, while the ZmMSI1 protein of *Z. mays* was only located in the nucleus [63]. Previous studies have illustrated that the OsFIE2 protein in *O. sativa* was located in both the nucleus and the cytoplasm [72], whereas the ZmFIE1 and ZmFIE2 proteins in *Z. mays* and the MhFIE protein in *M. hupehensis* were only localized to the nucleus [63,73].

The result of the chromosome location exhibited that 64 PcG genes in *M. truncatula* were unevenly distributed across the eight chromosomes, and the number of members of different components on the chromosomes was also different. For example, 15 of 27 *VRN1* genes were located on chromosome 1, and 20 *FIE* genes were unequally distributed on 7 chromosomes. Previous researches have illustrated that the *AtMEA* gene of *A. thaliana* was located on chromosome 1, and the *AtCLF* and *AtFIS2* genes were located on chromosome 2 [35,74]. Additionally, the *AtFIE* gene was located on chromosome 3 [37], the *AtVRN2* and *AtSWN* genes were located on chromosome 4 [75], and the *AtEMF1* and *AtEMF2* genes were located on chromosome 5 [27,76]. The *E(z)* genes of *T. aestivum* were distributed on chromosomes 4 and 7, the *Su(z)* genes were distributed on chromosomes 2 and 5, the *FIE* genes were distributed on chromosomes 4 and 7, the *MSI* and *BMI* genes were located on chromosome 5, the *LHP1* genes were located on chromosome 7, the *RING1* and *EMF1* genes were located on chromosome 3, and the *RING2* genes were located on chromosome 1 [62]. The *E(z)* genes of *H. vulgare* were located on chromosome 4, the *FIE* genes were located on chromosome 7, and the *Su(z)* genes were located on chromosomes 2 and 5 [77]. The *OsiEZ1* gene of *O. sativa indica* was located on chromosome 3; *OsCLF* of *O. sativa* was located on chromosome 6; *OsFIE1* and *OsFIE2* were located on chromosome 8; and *OsEMF2a* and *OsEMF2b* were, respectively, located on chromosome 4 and 9 [78]. Two copies of the *GmFIE* gene in *G. max* were located on chromosomes 2 and 10 [47]. These studies revealed that the distribution of PcG genes on chromosomes was quite different among species, but the chromosomal distribution of PcG genes in relative species had similar results. For instance, the *Su(z)* genes of *H. vulgare* and *T. aestivum* were both located on chromosomes 2 and 5 [62,77].

Gene duplication events usually include the segmental duplication and tandem duplication of the chromosome. This behavior can enhance the functional diversification of the gene family during plant evolution, which is necessary in order for plants to adapt to various environmental changes [79,80]. These two patterns of gene duplication can increase the number of members of the gene family [81]. Tandem duplication means that there had two or more homologous genes on the same chromosome located within a distance of 100 kb [82]. Segmental duplication means that DNA fragments greater than 1 Mb in length with a high sequence identity (>90%) usually map to two or more positions in the genome [83]. Recently, several studies have indicated that the partial PcG genes in cereals were produced by tandem replication events. For example, the *OsFIE1* and *OsFIE2* genes in *O. sativa* were derived from tandem replication events [78,84]. Among the *E(z)* and *Esc* genes of *Z. mays*, tandem duplication events also took place [63]. *ZCCT1* and *ZCCT2* of the VRN2 locus in diploid wheat were two tandem genes [85]. The *VRN2a* and *VRN2b* genes in hexaploid wheat were generated through tandem gene duplication [86]. More importantly, we observed some tandem duplication events in this study, which produced multiple copies of *VRN1*. Concurrently, five gene pairs were generated from segmental duplication events; it is tempting to speculate that the number of gene duplications was associated with the number of chromosomes and members of the family/subfamily.

Protein–protein interactions can reveal the regulatory relationships between proteins, which helps us to understand the potential function of these proteins in biochemical reactions [87]. In the present study, we predicted and analyzed the interactions between the 64 PcG proteins of *M. truncatula*, indicating that there were potential interactions between the PcG proteins in *M. truncatula*. A large number of reports demonstrated that a complex interaction network also existed among the PcG proteins of *A. thaliana* (Figure S6). On the one hand, these interactions included members of the same complex, such as the AtEMF1 and AtLHP1 proteins that interacted with the AtRING1A, AtRING1B, AtBMI1A, AtBMI1B, and AtBMI1C proteins in the PRC1 complex [16,67,88]. In the PRC2 complex, the AtFEI protein interacted with AtMEA, AtCLF, and AtMSI proteins [89,90,91,92,93]. On the other hand, these interactions also included members of different complexes, such as the AtRING1A and AtRING1B proteins of the PRC1 complex that interacted with the AtCLF protein of the PRC2 complex [67]. Simultaneously, the AtMSI and AtEMF2 proteins of the PRC2 complex interacted with the AtLHP1 protein of the PRC1 complex [94], and the AtMSI protein of the PRC2 complex interacted with the AtEMF1 protein of the PRC1 complex [23]. Furthermore, one protein may have interactions with other proteins that belong to different complexes at the same time. For instance, the AtRING1A protein of the PRC1 complex could interact with the AtCLF protein of the PRC2 complex and the AtLHP1 protein of the PRC1 complex [95]. Interestingly, a previous study found that PcG proteins of different species also interacted with each other, such as the MhFIE protein of *M. hupehensis* and the AtCLF protein of *A. thaliana* [73]. It is tempting to speculate that the protein interactions between PRC1 and PRC2 may be associated with the motif and domain characteristic of those proteins [6,96]. The results of this study illustrated that the MtMSI1;4 protein had a large number of interacting proteins, which was similar to what was observed in *A. thaliana* [96].

### 3.2. Evolutionary Relationship Analysis of PcG Proteins in M. truncatula

The phylogenetic analysis contributes to the comprehensive understanding of evolutionary relationships among family genes and reveals the phylogenetic relationships between different species [97,98]. According to the previous studies, *M. truncatula* underwent a whole-genome duplication (WGD) event about 58 million years ago and generated more gene pairs [99]. PcG genes have undergone multiple duplication events in the evolution of animals and plants [100]. The result of phylogenetic tree analysis in the present study demonstrated that the prominent evolutionary differences existed in the PcG family members between monocot and dicot plants. All PcG proteins were divided into three large groups based on complex to which they belonged and further clustered into nine subgroups according to their different components. It is noted that the VRN1 component was significantly different from the other four components in PRC1 as a result of VRN1 forming a new group on the phylogenetic tree. Simultaneously, several reports found that VRN1 acted downstream of VRN2-PRC2 to execute the PRC1-like function, while the mechanism of action was still unclear. Nevertheless, other components interacting with VRN1 have not yet been identified in the PRC1 complex; hence, some researchers thought that VRN1 was not the PRC1 core subunit in plants [20,101].

A previous study considered that RING1 has undergone one and two duplications in dicot and monocot ancestors, respectively [20]. Though RING1 proteins presented two copies in most species, *P. trichocarpa* and *Z. mays* possessed four copies and *B. rapa* had three copies [102]. We identified three copies of RING1 proteins in *M. truncatula*, supporting the idea that the duplication events of RING1 may occur after the divergence of the monocot and dicot plants [102]. Furthermore, the BMI1 orthologs in Brassicaceae, represented by *A. thaliana*, were divided into three groups corresponding to AtBMI1a, AtBMI1b, and AtBMI1c [103]. In this study, we identified only one BMI1 protein in *M. truncatula*, and the MtBMI1A protein had close evolutionary relationships with the corresponding protein in *G. max*. It was reported that LHP1 was a single copy in most plants, but there were multiple copies of LHP1 in *P. trichocarpa*, *G. max*, *M. domestica*, *B. napus*, and Solanaceae, and the orthologs of alfalfa LHP1 were absent, demonstrating that gene loss occurred at the same time as gene duplication [104]. We did not identify the orthologs of LHP1 in *M. truncatula*, which was consistent with the previous view that the gene only existed in the genome database of the Mt3.5v5 version [60]. A study found that, except for *C. sativus*, *G. raimondii*, *B. rapa*, and *Eutrema*, most species had a single homologous gene of EMF1, indicating that EMF1 was highly conserved in dicots [102]. In contrast, we identified two homologous genes of EMF1 in *M. truncatula*, and recent data showed that several EMF1 proteins were present in *T. aestivum* [62].

A previous study suggested that MSI1 proteins were likely to experience evolutionary divergence before the separation of monocots and dicots, and AtMSI2/AtMSI3 and AtMSI4/AtMSI5 formed similar protein pairs, illustrating that these proteins possessed redundant functions [105]. In the present study, we also observed that the MSI1 proteins of two dicots (*N. tomentosiformis* and *R. communis*) had close relatives in the monocots (*T. aestivum* and *O. sativa*). Although four MSI1 proteins were present in *M. truncatula*, these proteins did not form similar protein pairs. There is evidence that the common ancestor of CLF, SWN, and MEA underwent duplication events before the separation of monocot and dicot plants [35]. The relationships between SWN and CLF were closer than those of MEA, and although the SWN/CLF clades were more ancient than the MEA clades, SWN and CLF belonged to different clades [27,93]. The phylogenetic tree analysis in this study revealed that SWN and CLF were divided into different clades and then clustered into different sub-clades according to their monocots and dicots. Additionally, we observed that AtSWN was grouped into the clade including the SWN proteins of other dicot plants, which was consistent with the results of a previous study [78].

It was considered that *M. truncatula* had a closer phylogenetic relationship to *C. arietinum* on PcG proteins [57,58]. However, the present study suggested that more than one species had close evolutionary relationships with the PcG proteins in *M. truncatula*, prompting us to speculate that the number of species involved and analytical method used may have potential impacts on the result of the phylogenetic analysis. Previous reports have shown that the Su(z) and E(z) proteins in *T. aestivum* were, respectively, homologous to AtEMF2 and AtSWN/AtCLF [62]; the HvFIE protein was homologous to ZmFIE2 and OsnFIE2 [77]; and the VRN2 protein of *M. truncatula* was homologous to the VRN2 protein of *P. vulgaris* and *G. max* [39]. The phylogenetic results in this study were consistent with those of previous reports.

According to the results of the syntenic analysis, we found that the PcG genes in dicot plants were more conserved when compared with monocots. *M. truncatula* had a closer evolutionary relationship to *G. max* in terms of its extent of PcG genes, while it was distant from monocots (*B. distachyon*, *O. sativa*, and *S. bicolor*). In addition, five genes, including *MtBMI1A*, *MtSWN*, *MtMEA;2*, *MtFIE;1*, and *MtMSI1;3*, had collinearity relationships with other six species, indicating that these genes may be ancestral genes. There have only been a few studies on the syntenic analysis of PcG genes in other species. A previous study demonstrated that *HvFIE*, *HvE(z)*, *HvEMF2a*, and *HvEMF2b* of *H. vulgare* had collinearity relationships with *OsnFIE2*, *OsiEZ1*, *OsEMF*, and *OsVEF* of *O. sativa nipponbare*, respectively [78].

### 3.3. Expression Pattern Analysis of PcG Genes in M. truncatula

Promoters contain important regions that regulate gene expression. The response patterns of the target genes to the external environment can be predicted by analyzing the types of cis-acting elements involved [106,107]. To further study the regulatory mechanism of *M. truncatula* PcG genes in the processes of growth, development, and environmental stress, we predicted and analyzed the cis-acting elements in the promoter region. The results of this study revealed that the cis-acting elements of *M. truncatula* PcG genes could respond to various environmental factors (drought, low temperature, circadian rhythm, light, and wound) and plant hormones (ABA and IAA). Accordingly, we speculated that these cis-acting elements may play a functional role in the regulatory pathways of stress response and development in *M. truncatula*. Gene expression patterns can provide important information for gene functions [108]. Further analysis of the expression changes in PcG genes in *M. truncatula* could verify the functions of the cis-acting elements of these genes, contributing to understanding the potential roles of PcG genes in the different developmental stages, tissues, and environmental stresses of *M. truncatula*.

In this study, we combined microarray expression data, RNA-seq data, and qRT-PCR data to analyze the expression patterns of PcG genes in *M. truncatula*. Among them, the average expression levels of the two *MSI* genes (*MtMSI1;4* and *MtMSI1;1*) in different tissues and different treatments were higher than those of other genes in microarray expression data, which was consistent with previous studies in *A. thaliana* and *Lycopersicon esculentum* [105,109]. Meanwhile, the results of this study illustrated that the *MtMSI1;4* gene (homologous to *AtMSI*) exhibited significant expression changes in different tissues and treatment conditions. For example, the *MtMSI1;4* gene had high expression levels in pollinated seeds and 7, 10, and 14 days after drought treatment, but had low expression levels 2 and 24 h after MeJA treatment. As mentioned above, the MeJA responsive elements (CGTCA-motif and TGACG-motif) and ABA responsive elements (ABRE) existed in the promoter region of the *MtMSI1;4* gene (Table S7). Hence, we speculated that this gene may be involved in the regulation of the seed development of *M. truncatula* and had a response to drought stress and MeJA. Similar results were also found in *A. thaliana*. *AtMSI1* had a significant effect on plant development, and the mutants would lead to a loss of flower morphologies and the destruction of ovules [105].

In the RNA-seq data of different tissues, the expression levels of *MtVRN1;4* in each tissue were both higher than those in other genes. Simultaneously, the expression levels of this gene in the aboveground parts of *M. truncatula* were significantly higher than those in the underground parts. We also found that there were a large number of light-responsive elements (such as Box 4, G-Box, TCT-motif, etc.) in the promoter sequence of the *MtVRN1;4* gene, suggesting that the gene may be involved in the photomorphogenesis of the aboveground parts of *M. truncatula*. The five *VRN1* genes (*MtVRN1;4*, *MtVRN1;17*, *MtVRN1;20*, *MtVRN1;22*, *MtVRN1;23*), *MtEMF2*, and *MtRING1A* had high expression levels in flowers and their promoter sequences contained light-responsive elements (Box 4 and TCT-motif), indicating that these genes may be involved in the flowering regulatory pathways of *M. truncatula*. Similar results were observed in research on the *AtRING1A* gene in *A. thaliana* [110]. Moreover, the *MtVRN1;4* and *MtVRN1;22* genes were highly expressed in seedlings, and their promoter sequences contained seed-specific regulatory elements (RY-element), meristem expression regulatory elements (CAT-box), and endosperm expression regulatory elements (GCN4_motif). These genes may be involved in the process of seed germination and meristem growth. Similar results were found in a previous study of *AtVRN1* gene function [24]. The results from the RNA-seq data of Nod factor and mock treatment indicated the expression levels of *MtCLF* and two *MSI* genes (*MtMSI1;4* and *MtMSI1;5*) had obvious changes. A previous study illustrated that *AtCLF* played an important role in maintaining the activity of root meristems [32], and that *AtMSI1* was required for the maintenance of shoot and root apical meristems [42]. Hence, we speculated that these genes were involved in the growth and development of the *M. truncatula* roots under the two treatment conditions.

More importantly, we found some similar expression patterns in the two RNA-seq data of different stress treatments. For instance, *MtFIE;14* had the highest expression level under cold stress as the result of the gene promoter sequence containing defense and stress responsive elements (TC-rich repeats). *MtFIE;11* had a high expression level under drought stress, which was related to the gene promoter sequence containing drought-inducing elements (MBS). *MtFIE;4* had a high expression level under cold stress and freezing stress, since the gene promoter sequence contained defense and stress responsive elements (TC-rich repeats) and low-temperature responsive elements (LTR). Concurrently, we identified many DEGs related to multiple stress responses through the differential gene expression analysis, and the results suggested that different genes may have different responsive and regulatory pathways. The co-expression network and enrichment analysis also provided more information about the *M. truncatula* PcG genes participating in specific biological processes in a co-regulated manner. Three regulatory networks with *MtFIE;17*, *MtVRN1;3*, and *MtCLF* as the core genes may also play different roles in growth, development, and adaptability to the environment.

In addition, we analyzed the expression patterns of PcG genes using qRT-PCR technology in different tissues, developmental stages, and treatment conditions. The results illustrated that *MtBMI1A*, *MtFIE;2*, *MtMEA;1*, *MtRING1A*, and *MtSWN* possessed similar expression patterns in different tissues and developmental stages. These genes had the highest relative expression levels in leaves and fruits, as well as leaves at 14 and 63 days after germination; hence, we speculated that those genes may participate in the seed development and leaf growth of *M. truncatula*. A large number of previous reports provided references for the abovementioned results. For example, the *AtBMI1* gene was necessary to maintain cell differentiation [111], *AtBMI1a* and *AtBMI1b* regulated fruit development by controlling the expression of embryonic stem cell regulatory factors [88], and the *AtBMI1c* gene was expressed in endosperm and stamen [112]. *FIE* was involved in a series of growth and development processes, including flowering, early seed development, dormancy, seed germination, and seedling development in *A. thaliana* [45,84,92]. We also found that the *MtEMF1;1* and *MtMSI1;4* genes had similar expression patterns. The relative expression levels of these genes gradually increased after seed germination, and they had high relative expression levels in flowers and leaves, illustrating that these genes may be involved in the regulation of flowering and leaf growth. These results were consistent with previous reports that *MSI1* was considered to be necessary for regulating flowering time [48,113], and that *AtEMF1* played an important role in vegetative growth and flowering regulation [4,17,76,114].

Under two different stresses and two hormone treatments, the expression patterns of the same gene were found to be different among different treatments, but the results displayed that the PcG genes produced obvious impacts on drought, low temperatures, ABA, and MeJA. These genes promoter sequences contained cis-acting elements related to environmental stimuli, such as drought responsive elements (MBS), low-temperature responsive elements (LTR), ABA-responsive elements (ABRE), MeJA-responsive elements (CGTCA-motif and TGACG-motif), defense- and stress-responsive elements (TC-rich repeats), etc. Numerous studies have demonstrated that, in addition to regulating plant growth and development, PcG genes also respond to environmental changes. For instance, the *AtBMI1a*/*1b* and *AtMSI1* genes participated in drought response [9,115] and the *GmFIE* gene was involved in cold and drought stress [47]. *EMF1* and *EMF2* regulated multiple biological processes, including photosynthesis, seed development, phytohormone, stress, and cold signals [54]. In addition to playing an essential role in regulating the vernalization and photoperiod response of *T. aestivum*, *VRN2* also had regulatory functions in dehydration, wound, heat shock, and ABA [40]. These studies suggested that PcG genes perform crucial functions in the adaptation of plants to the external environment.

Recently, numerous studies and reports on the gene family of *M. truncatula* have been carried out. However, different versions of the genome database may have an impact on the number of gene family members. Pecrix et al. completed the whole-genome sequencing of *M. truncatula* A17 based on PacBio technology and also carried out the genome-wide detection of histone markers [116]. This is very important for the research on the biological and epigenetic characteristics of *M. truncatula* based on the whole genome.

## 4. Materials and Methods

### 4.1. Materials, Growth Conditions, Treatment, and Sampling

*M. truncatula* (cv. Jemalong A17) seeds were sterilized and placed in the sterile Murashige and Skoog (MS) solid medium (the seeds were first treated with sulfuric acid for 10 min to break the seed coat and then sterilized in 1% NaClO solution for 10 min). After 7 days of vernalization at 4 °C, they were placed in the growth chambers (16/8 h light/dark photoperiod, 24 °C/24 °C day/night temperature, 30% relative humidity). After germination for 14 days, materials were transplanted to plastic pots (vermiculite/peat soil = 3:1) in a greenhouse for growth (16/8 h light/dark photoperiod, 25 °C/23 °C day/night temperature, 52% relative humidity).

We performed all of the treatments when the materials germinated for 6 weeks. For drought stress, plants were irrigated with 300 mmol/L mannitol solution. For cold stress, plants were transferred to a growth chamber at 4 °C. For phytohormone treatments, plants were treated with 100 μmol/L abscisic acids (ABA) and 100 μmol/L methyl jasmonate (MeJA), respectively. Samples were collected from seedling leaves at four time points (0, 3, 12, and 24 h), including three biological replicates, and the samples treated for 0 h were used as controls. All of the samples were immediately frozen in nitrogen and stored at −80 °C.

### 4.2. Download and Analysis of Genome Data

The plant genome data and annotation files used in this study were downloaded from the Ensembl Plants Database (http://plants.ensembl.org/index.html (accessed on 3 March 2021)), and we used the TBtools (v1.055) software (https://github.com/CJ-Chen/TBtools, accessed on 3 March 2021) to obtain the promoter, mRNA, CDS, and protein sequences [117].

### 4.3. Identification of PcG Genes in M. truncatula

The amino acid sequences of *A. thaliana* PcG proteins were collected from the TAIR database (https://www.arabidopsis.org/ (accessed on 3 March 2021)). Concurrently, using *A. thaliana* PcG protein sequences as the query object, we used the TBtools software and online analysis website (http://www.ncbi.nlm.nih.gov/BLAST (accessed on 3 March 2021)) to conduct a BLASTP search against the *M. truncatula* genome database. Finally, the Batch web CD search tool (https://www.ncbi.nlm.nih.gov/Structure/bwrpsb/bwrps-b.cgi (accessed on 3 March 2021)) was used to further confirm the integrity of the conserved domains of candidate PcG members to screen *M. truncatula* PcG members. These genes were named according to the gene locations on the chromosomes and the components to which they belonged [118].

### 4.4. Basic Analysis of PcG Proteins

The MW, pI, and GRAVY of *M. truncatula* PcG proteins were analyzed using the ProtParam ExPASy server (http://www.expasy.org/tools/ (accessed on 6 March 2021)). Moreover, we predicted the subcellular locations of *M. truncatula* PcG proteins using WoLF PSORT (https://wolfpsort.hgc.jp/ (accessed on 6 March 2021)) and Plant-mPLoc (http://www.csbio.sjtu.edu.cn/bioinf/plant-multi/ (accessed on 6 March 2021)). The secondary and tertiary structures of *M. truncatula* PcG proteins were analyzed through SOPMA (https://npsa-prabi.ibcp.fr/cgi-bin/npsa_automat.pl?page=npsa_sopma.html (accessed on 9 March 2021)) and Phyre v2.0 (http://www.sbg.bio.ic.ac.uk/phyre2/html/page.cgi?id=index (accessed on 9 March 2021)), respectively.

### 4.5. Sequence Analysis and Chromosome Location

We identified the conserved motifs of *M. truncatula* PcG genes using the MEME tool (http://meme-suite.org/tools/meme (accessed on 10 March 2021)) and predicted the cis-acting elements in the promoter sequences of those genes via PlantCARE (http://bioinformatics.psb.ugent.be/webtools/plantcare/html/ (accessed on 12 March 2021)). The conserved domain information of PcG genes was obtained from the Batch web CD search tool (https://www.ncbi.nlm.nih.gov/Structure/bwrpsb/bwrpsb.cgi (accessed on 15 March 2021)). Meanwhile, the TBtools software was used to analyze the gene structure, chromosome location, and visual display.

### 4.6. Prediction of Protein Interaction Networks

We predicted the protein interaction networks of *M. truncatula* PcG proteins through the STRING v11.0 program (https://www.string-db.org/ (accessed on 15 March 2021)). The confidence parameters (combined score) were set at a 0.40 threshold, and the ‘Network type’ was ‘full network (the edges indicate both functional and physical protein associations)’. The results were visualized using the Cytoscape v3.8.2 software (https://cytoscape.org/download.html (accessed on 3 March 2021)).

### 4.7. Phylogenetic and Synteny Analysis

We constructed an unrooted phylogenetic tree was constructed with the PcG protein sequences of *M. truncatula* and 26 other species using the MEGA-X software (https://www.megasoftware.net/download_form (accessed on 3 March 2021)) with the maximum likelihood method. Furthermore, we analyzed the syntenic relationships of the PcG family genes between the *M. truncatula*, *A. thaliana*, *Sorghum bicolor*, *Brachypodium distachyon*, *Oryza sativa*, *Glycine max*, and *Phaseolus vulgaris* genomes using the TBtools software, then obtained the syntenic block data from the analysis results [119].

### 4.8. PcG Genes Expression Analysis

In order to understand the expression patterns of *M. truncatula* PcG genes in different developmental stages, tissues, and treatments, we collected and analyzed the microarray expression data and transcriptome sequencing data of *M. truncatula*. Among them, the microarray and RNA-seq data (Table S8) were downloaded from the online database (https://mtgea.noble.org/v3/ (accessed on 20 March 2021)) and the NCBI website (https://www.ncbi.nlm.nih.gov/sra/?term= (accessed on 20 March 2021 and 27 June 2021)), respectively. After screening the expression data of *M. truncatula* PcG genes under different developmental stages, tissues, stresses, and phytohormone treatments, the expression values were log2-normalized and visualized for heat maps using the TBtools software. We analyzed the count matrix of RNA-seq data using the edgeR package in R software v3.6.3 (https://cran.r-project.org/bin/windows/base/old/3.6.3/ (accessed on 9 July 2020)), since there were no biological replicates in the sequencing samples. In addition, the paired samples were standardized through the TMM method and the differentially expressed genes (DEGs) were identified (FDR < 0.05, |Log2FC| > 1). Ultimately, we generated a volcano map of the DEGs using the EnhancedVolcano package in R software v4.0.3 (https://cran.r-project.org/bin/windows/base/old/4.0.3/ (accessed on 13 November 2020)).

### 4.9. Co-Expression and GO Enrichment Analysis of PcG Genes

We integrated the microarray and RNA-seq data and constructed the co-expression network as follows: firstly, the gene expression values from a total of 70 samples were log2 transformed and normalized by z-score transformation. Then, the expression correlation of the PcG genes was calculated by the Pearson correlation coefficient (PCC) and co-expressed gene pairs were screened with a cut-off PCC value > 0.8 [120,121]. The network was visualized and analyzed by Cytoscape. Moreover, the Molecular Complex Detection (MCODE) plugin in Cytoscape was used to screen modules of the co-expression network (degree cutoff = 2, node score cutoff = 0.2, k-core = 2, max. depth = 100). To better understand the potential biological mechanisms related to the co-expression network, a GO enrichment analysis was performed using the agriGO website (http://systemsbiology.cau.edu.cn/agriGOv2/index.php (accessed on 27 June 2021)) to identify the significantly enriched (*p* value < 0.05) GO terms of the top two MCODE genes (the number of genes in MCODE-3 is less than 10).

### 4.10. RNA Extraction and qRT-PCR Analysis

We detected the relative expression levels of the *M. truncatula* PcG genes identified in this study through qRT-PCR analysis. RNA extraction was performed using the Plant Total RNA Kit (Sangon Biotech, Shanghai, China) according to the manufacturer’s instructions. The first-strand cDNA was synthesized with the M-MLV cDNA synthesis kit (Sangon Biotech, Shanghai, China) according to the manufacturer’s protocol. The primers of reference gene *MtActin* and 12 representative PcG genes (these genes were most similar to the corresponding genes of *A. thaliana*) for qRT-PCR analysis were designed by the Primer Premier 6.0 (http://www.premierbiosoft.com/ (accessed on 16 March 2021)) software (Table S9). The RT-qPCR was performed on the StepOnePlus real-time PCR system (Applied Biosystems, Foster City, CA, USA) using a 2xSG Fast qPCR Master Mix (Low Rox) (Sangon Biotech, Shanghai, China). The relative expression levels of the PcG genes were calculated using the 2^−ΔΔCt^ method, and the results were visualized by the GraphPad Prism v8.0.2 software (https://www.graphpad.com/ (accessed on 15 November 2019)).

## 5. Conclusions

In this work, we performed the first comprehensive and systematic analysis of the *M. truncatula* PcG proteins, which well elucidated the fundamental characteristics of the physics, chemistry, and biology of these members. We found an irregular chromosome distribution of the 64 *M. truncatula* PcG members, and they showed complex protein interactions. Phylogenetic tree and synteny analyses provided new insights into the evolutionary characteristics of *M. truncatula* PcG members. Expression pattern analysis revealed the potential function of *M. truncatula* PcG genes in regulating the growth and development of *M. truncatula* as well as responding to various environmental conditions. Moreover, the results of the present study produced scientific evidence for further research on the regulatory mechanism and functional role of PcG proteins in other legumes.

## Figures and Tables

**Table 1:** Basic information of PcG genes in *M. truncatula*.

Gene Name	Gene ID	Location	ORF ^1^ Length (bp)	Number of Exons	MW ^2^ (kDa)	pI ^3^ Value	GRAVY ^4^
*MtBMI1A*	Medtr7g096210.1	38,583,482–38,583,911	1290	7	47.37	9.24	−0.576
*MtEMF1;1*	Medtr3g110082.1	50,968,972–50,970,140	3507	7	129.51	8.92	−0.826
*MtEMF1;2*	Medtr3g110132.1	51,072,108–51,073,292	3555	5	131.39	9.02	−0.839
*MtRING1A*	Medtr2g090595.1	38,732,918–38,733,422	1515	10	58.11	4.77	−1.259
*MtRING1B;1*	Medtr4g088520.1	35,141,049–35,141,489	1323	10	49.82	5.05	−0.961
*MtRING1B;2*	Medtr4g091110.1	36,070,448–36,070,700	759	6	29.13	3.97	−1.125
*MtVRN1;1*	Medtr1g021270.1	6,335,039–6,335,635	1791	6	66.67	8.79	−0.379
*MtVRN1;2*	Medtr1g021290.1	21,113,921–21,114,394	1044	3	39.36	6.56	−0.501
*MtVRN1;3*	Medtr1g021320.1	6,356,167–6,357,009	2529	8	94.10	8.70	−0.476
*MtVRN1;4*	Medtr1g021330.1	6,366,047–6,366,432	1158	4	43.38	8.67	−0.434
*MtVRN1;5*	Medtr1g021360.1	6,377,524–6,378,002	1437	5	54.49	8.91	−0.542
*MtVRN1;6*	Medtr1g021380.1	6,390,073–6,390,423	1053	4	39.57	6.83	−0.426
*MtVRN1;7*	Medtr1g021400.1	6,397,039–6,397,407	1107	4	41.12	8.52	−0.516
*MtVRN1;8*	Medtr1g021410.1	6,403,159–6,403,520	1086	4	41.12	9.10	−0.615
*MtVRN1;9*	Medtr1g021435.1	6,414,120–6,414,492	1119	4	41.83	8.78	−0.430
*MtVRN1;10*	Medtr1g021440.1	6,419,474–6,419,873	1200	4	44.91	8.06	−0.594
*MtVRN1;11*	Medtr1g021500.1	6,438,573–6,439,064	1476	6	54.50	8.78	−0.617
*MtVRN1;12*	Medtr1g034210.1	12,425,305–12,425,675	1113	5	42.30	8.38	−0.699
*MtVRN1;13*	Medtr1g034240.1	12,442,470–12,442,903	1302	4	49.15	8.93	−0.577
*MtVRN1;14*	Medtr1g035460.1	12,873,695–12,874,091	1191	6	46.28	8.68	−0.842
*MtVRN1;15*	Medtr1g108780.1	49,164,002–49,164,463	1386	6	53.61	8.73	−0.666
*MtVRN1;16*	Medtr3g005420.1	198,959–199,501	1629	6	62.39	9.33	−0.544
*MtVRN1;17*	Medtr3g061320.1	24,354,366–24,354,645	840	4	32.68	9.69	−0.510
*MtVRN1;18*	Medtr3g098790.1	45,269,198–45,269,526	987	4	38.00	9.87	−0.427
*MtVRN1;19*	Medtr4g068320.1	25,619,387–25,619,816	1290	4	48.52	8.75	−0.536
*MtVRN1;20*	Medtr5g070440.1	29,839,127–29,839,401	825	3	31.93	9.54	−0.570
*MtVRN1;21*	Medtr6g061070.1	21,113,921–21,114,394	1422	7	54.38	6.84	−0.642
*MtVRN1;22*	Medtr7g050560.1	16,862,100–16,862,415	948	5	36.27	9.70	−0.387
*MtVRN1;23*	Medtr7g050580.1	16,867,490–16,867,805	315	5	36.08	9.72	−0.610
*MtVRN1;24*	Medtr7g050590.1	16,873,037–16,873,334	894	3	34.41	9.99	−0.631
*MtVRN1;25*	Medtr7g050710.1	17,605,025–17,605,349	975	4	37.06	9.91	−0.544
*MtVRN1;26*	Medtr7g050720.1	17,612,279–17,612,599	963	4	36.77	9.50	−0.457
*MtVRN1;27*	Medtr8g103940.1	43,749,832–43,750,330	1497	7	57.36	9.15	−0.679
*MtSWN*	Medtr1g086980.1	38,931,514–38,932,145	2496	17	93.50	7.97	−0.775
*MtCLF*	Medtr5g016870.1	6,095,959–6,096,822	2592	18	96.55	8.60	−0.819
*MtMEA;1*	Medtr7g055660.1	19,084,301–19,085,033	2199	16	84.19	8.18	−0.799
*MtMEA;2*	Medtr7g109560.1	44,830,300–44,831,064	2295	16	87.40	7.23	−0.813
*MtEMF2*	Medtr1g090240.1	40,419,285–40,419,977	2079	17	77.93	6.41	−0.348
*MtVRN2*	Medtr5g013150.1	4,143,460–4,143,938	1437	16	54.56	8.30	−0.567
*MtFIE;1*	Medtr1g011610.2	2,109,762–2,110,664	2709	18	99.01	6.45	−0.695
*MtFIE;2*	Medtr1g028310.1	9,510,205–9,510,577	1119	13	41.61	6.21	−0.119
*MtFIE;3*	Medtr1g072140.1	32,005,164–32,005,480	951	2	34.85	8.69	−0.275
*MtFIE;4*	Medtr1g090827.1	40,854,644–40,855,860	3651	3	136.27	6.45	−0.186
*MtFIE;5*	Medtr2g436510.1	14,197,679–14,198,159	1443	17	53.14	9.23	−0.485
*MtFIE;6*	Medtr2g086140.1	36,225,284–36,226,276	2979	10	108.23	8.04	−0.328
*MtFIE;7*	Medtr2g102267.1	44,039,290–44,039,607	954	10	36.20	5.31	−0.348
*MtFIE;8*	Medtr2g102277.2	44,045,802–44,046,723	2766	24	104.06	4.93	−0.292
*MtFIE;9*	Medtr3g061450.1	24,506,254–24,506,567	942	11	35.17	6.34	−0.383
*MtFIE;10*	Medtr3g106080.1	48,932,864–48,933,533	2010	19	74.24	6.50	−0.438
*MtFIE;11*	Medtr3g116500.1	54,500,761–54,501,139	1137	6	41.04	7.58	−0.217
*MtFIE;12*	Medtr4g011950.1	3,145,419–3,146,350	2796	24	105.08	4.87	−0.327
*MtFIE;13*	Medtr4g053675.1	19,445,007–19,446,038	3096	10	112.64	6.62	−0.358
*MtFIE;14*	Medtr4g071130.1	26,806,737–26,807,943	3621	4	135.47	6.49	−0.221
*MtFIE;15*	Medtr4g095058.1	39,607,610–39,608,188	1737	14	64.92	6.39	−0.595
*MtFIE;16*	Medtr4g119620.1	49,575,751–49,576,077	981	2	35.88	6.78	−0.350
*MtFIE;17*	Medtr4g125710.1	52,171,088–52,171,409	966	2	34.39	5.55	−0.075
*MtFIE;18*	Medtr5g022690.1	8,972,131–8,972,619	1467	5	55.02	6.08	−0.646
*MtFIE;19*	Medtr6g004040.1	91,010–91,770	2283	18	83.11	7.93	−0.306
*MtFIE;20*	Medtr7g058460.1	21,059,701–21,060,485	2355	17	85.49	6.36	−0.566
*MtMSI1;1*	Medtr2g039250.1	17,196,265–17,196,765	1503	15	55.61	6.11	−0.627
*MtMSI1;2*	Medtr2g100090.1	42,972,259–42,972,738	1440	12	52.71	5.26	−0.406
*MtMSI1;3*	Medtr4g073080.1	27,640,107–27,640,512	1218	6	45.75	4.66	−0.536
*MtMSI1;4*	Medtr4g096880.1	38,572,495–38,572,918	1272	5	48.22	4.75	−0.569
*MtMSI1;5*	Medtr8g080700.1	34,739,363–34,739,832	1410	12	52.04	4.97	−0.586

Abbreviation: ^1^ ORF, open reading frame; ^2^ MW, molecular weights; ^3^ pI, isoelectric points; ^4^ GRAVY, grand average of hydropathicity.

**Table 2:** Prediction of protein secondary structure and subcellular location of *M. truncatula* PcG proteins.

Gene Name	Protein Secondary Structure				Subcellular Location Prediction	
	α-Helix (%)	Extended Strand (%)	Β-Sheet (%)	*Random coil* (%)	Plant-mPLoc	WoLF PSORT
*MtBMI1A*	31.24	7.93	2.10	58.74	Nucleus	Nuclear
*MtEMF1;1*	17.64	7.45	1.71	73.20	Nucleus	Nuclear
*MtEMF1;2*	18.58	9.29	2.28	69.85	Nucleus	Nuclear
*MtRING1A*	44.64	7.34	2.98	45.04	Nucleus	Nuclear
*MtRING1B;1*	40.91	7.95	2.73	48.41	Nucleus	Nuclear
*MtRING1B;2*	50.79	11.90	5.16	32.14	Nucleus	Nuclear
*MtVRN1;1*	20.81	20.97	5.54	52.68	Chloroplast/Nucleus	Nuclear
*MtVRN1;2*	24.50	23.05	4.32	48.13	Cell wall/Nucleus	Nuclear
*MtVRN1;3*	28.74	18.76	5.23	47.27	Nucleus	Nuclear
*MtVRN1;4*	22.60	16.36	4.16	56.88	Chloroplast/Nucleus	Nuclear
*MtVRN1;5*	24.48	23.01	6.07	46.44	Chloroplast/Nucleus	Nuclear
*MtVRN1;6*	19.71	20.29	6.86	53.14	Nucleus	Nuclear
*MtVRN1;7*	19.02	22.01	5.71	53.26	Cell wall/Nucleus	Nuclear
*MtVRN1;8*	18.28	22.99	4.16	54.57	Cell wall/Nucleus	Nuclear
*MtVRN1;9*	17.74	20.70	6.72	54.84	Cell wall/Nucleus	Nuclear
*MtVRN1;10*	20.55	18.30	4.76	56.39	Nucleus	Nuclear
*MtVRN1;11*	24.85	16.29	2.65	56.21	Nucleus	Nuclear
*MtVRN1;12*	22.16	20.27	10.00	47.57	Nucleus	Chloroplast
*MtVRN1;13*	20.32	18.94	6.70	54.04	Nucleus	Chloroplast
*MtVRN1;14*	15.40	21.46	5.30	57.83	Nucleus	Nuclear
*MtVRN1;15*	14.75	22.34	6.07	56.83	Nucleus	Nuclear
*MtVRN1;16*	22.14	23.43	5.90	48.52	Nucleus	Chloroplast
*MtVRN1;17*	14.70	25.45	9.32	50.54	Chloroplast/Nucleus	Chloroplast
*MtVRN1;18*	13.41	24.39	5.79	56.40	Chloroplast/Nucleus	Nuclear
*MtVRN1;19*	22.84	19.11	6.29	51.75	Chloroplast/Nucleus	Nuclear
*MtVRN1;20*	17.88	27.74	7.30	47.08	Nucleus	Nuclear
*MtVRN1;21*	15.01	23.89	7.19	53.91	Nucleus	Nuclear
*MtVRN1;22*	13.33	23.49	8.25	54.92	Chloroplast/Nucleus	Cytoplasmic
*MtVRN1;23*	15.24	25.08	7.62	52.06	Chloroplast/Nucleus	Nuclear
*MtVRN1;24*	15.49	24.24	7.07	53.20	Nucleus	Nuclear
*MtVRN1;25*	14.20	23.77	6.79	55.25	Nucleus	Nuclear
*MtVRN1;26*	13.75	24.69	9.06	52.50	Nucleus	Cytoplasmic
*MtVRN1;27*	14.66	21.89	5.62	57.83	Nucleus	Nuclear
*MtSWN*	29.96	10.71	5.90	53.43	Nucleus	Nuclear
*MtCLF*	30.94	10.43	4.98	53.65	Nucleus	Nuclear
*MtMEA;1*	32.24	12.02	4.10	51.64	Nucleus	Nuclear
*MtMEA;2*	31.94	13.22	3.27	51.57	Nucleus	Nuclear
*MtEMF2*	26.59	21.39	5.78	46.24	Nucleus	Nuclear
*MtVRN2*	33.05	12.13	5.02	49.79	Nucleus	Nuclear
*MtFIE;1*	29.82	16.63	7.32	46.23	Nucleus	Nuclear
*MtFIE;2*	10.48	35.22	11.29	43.01	Nucleus	Cytoplasmic
*MtFIE;3*	6.01	44.94	13.61	35.44	Nucleus	Chloroplast
*MtFIE;4*	32.07	23.60	8.80	35.53	Nucleus	Nuclear
*MtFIE;5*	11.04	31.67	9.17	48.12	Nucleus	Nuclear
*MtFIE;6*	18.65	22.98	6.35	52.02	Nucleus	Nuclear
*MtFIE;7*	12.62	41.64	14.20	31.55	Nucleus	Nuclear
*MtFIE;8*	35.40	25.95	9.66	28.99	Nucleus	Cytoplasmic
*MtFIE;9*	7.35	38.98	9.90	43.77	Nucleus	Cytoplasmic
*MtFIE;10*	31.84	23.17	6.28	38.71	Nucleus	Nuclear
*MtFIE;11*	15.34	36.51	11.38	36.77	Endoplasmic reticulum	Nuclear
*MtFIE;12*	34.16	25.99	10.42	29.43	Endoplasmic reticulum/Golgi apparatus	Chloroplast
*MtFIE;13*	20.76	21.34	5.92	51.99	Nucleus	Nuclear
*MtFIE;14*	31.51	23.71	8.71	36.07	Nucleus	Chloroplast
*MtFIE;15*	17.82	29.58	9.34	43.25	Nucleus	Nuclear
*MtFIE;16*	3.68	45.09	14.42	36.81	Nucleus	Nuclear
*MtFIE;17*	10.59	40.50	12.46	36.45	Nucleus	Cytoplasmic
*MtFIE;18*	32.38	28.89	10.45	28.28	Nucleus	Nuclear
*MtFIE;19*	26.32	19.74	6.32	47.63	Nucleus	Chloroplast
*MtFIE;20*	28.19	17.22	5.74	48.85	Nucleus	Nuclear
*MtMSI1;1*	16.40	26.00	5.00	52.60	Nucleus	Nuclear
*MtMSI1;2*	12.94	28.60	2.92	55.53	Nucleus	Cytoplasmic
*MtMSI1;3*	12.59	30.62	5.93	50.86	Nucleus	Nuclear
*MtMSI1;4*	14.89	29.79	5.67	49.65	Nucleus	Nuclear
*MtMSI1;5*	16.20	24.00	5.54	54.16	Nucleus	Mitochondrial

## Data Availability

All relevant data are contained within the paper and Supplementary Files.

## Supplementary Materials

The following are available online at https://www.mdpi.com/article/10.3390/ijms22147537/s1: Figure S1: The prediction of tertiary structure of PRC1 proteins in *M. truncatula*; Figure S2: The prediction of tertiary structure of PRC2 proteins in *M. truncatula*; Figure S3: The motif logo analysis of PRC1; Figure S4: Statistics of cis-acting elements of PRC1 (A) and PRC2 (B) in *M. truncatula*; Figure S5: The motif logo analysis of PRC2; Figure S6: Prediction of protein–protein interaction among PcG members in *A. thaliana*; Figure S7-1: Multiple sequence alignment of BMI1 proteins between *M. truncatula* and *A. thaliana*; Figure S7-2: Multiple sequence alignment of EMF1 proteins between *M. truncatula* and *A. thaliana*; Figure S7-3: Multiple sequence alignment of RING1 proteins between *M. truncatula* and *A. thaliana*; Figure S7-4: Multiple sequence alignment of VRN1 proteins between *M. truncatula* and *A. thaliana*; Figure S7-5: Multiple sequence alignment of SWN, CLF, and MEA proteins between *M. truncatula* and *A. thaliana*; Figure S7-6: Multiple sequence alignment of EMF2 and VRN2 proteins between *M. truncatula* and *A. thaliana*; Figure S7-7: Multiple sequence alignment of FIE proteins between *M. truncatula* and *A. thaliana*; Figure S7-8: Multiple sequence alignment of MSI1 proteins between *M. truncatula* and *A. thaliana*; Figure S8: Identification of differentially expressed genes of PcG members based on RNA-seq data in different tissues; Table S1-1: The tandem duplication genes and segmental duplication genes of whole genome in *M. truncatula*; Table S1-2: The tandem duplication genes and segmental duplication genes of PcG family in *M. truncatula*; Table S2: List of protein–protein interaction among PcG members in *M. truncatula*; Table S3: Syntenic relationships between *M. truncatula* and other six plant species; Table S4: Statistics of syntenic blocks among six plant species; Table S5: Enrichment analysis of top two MCODE genes function; Table S6: Gene information of different differentially expressed gene sets; Table S7: Statistics of cis-acting elements of PRC1 and PRC2 members in *M. truncatula*; Table S8: *M. truncatula* RNA-seq data used in this study; Table S9: Primer information of qRT-PCR.
