# Supplementary figures and images for: Genome-Wide Identification and Analysis of the Polycomb Group Family in Medicago truncatula
Source: Int J Mol Sci. 2021 Jul 14;22(14):7537. doi: 10.3390/ijms22147537 (PMC8303337; doi:10.3390/ijms22147537)

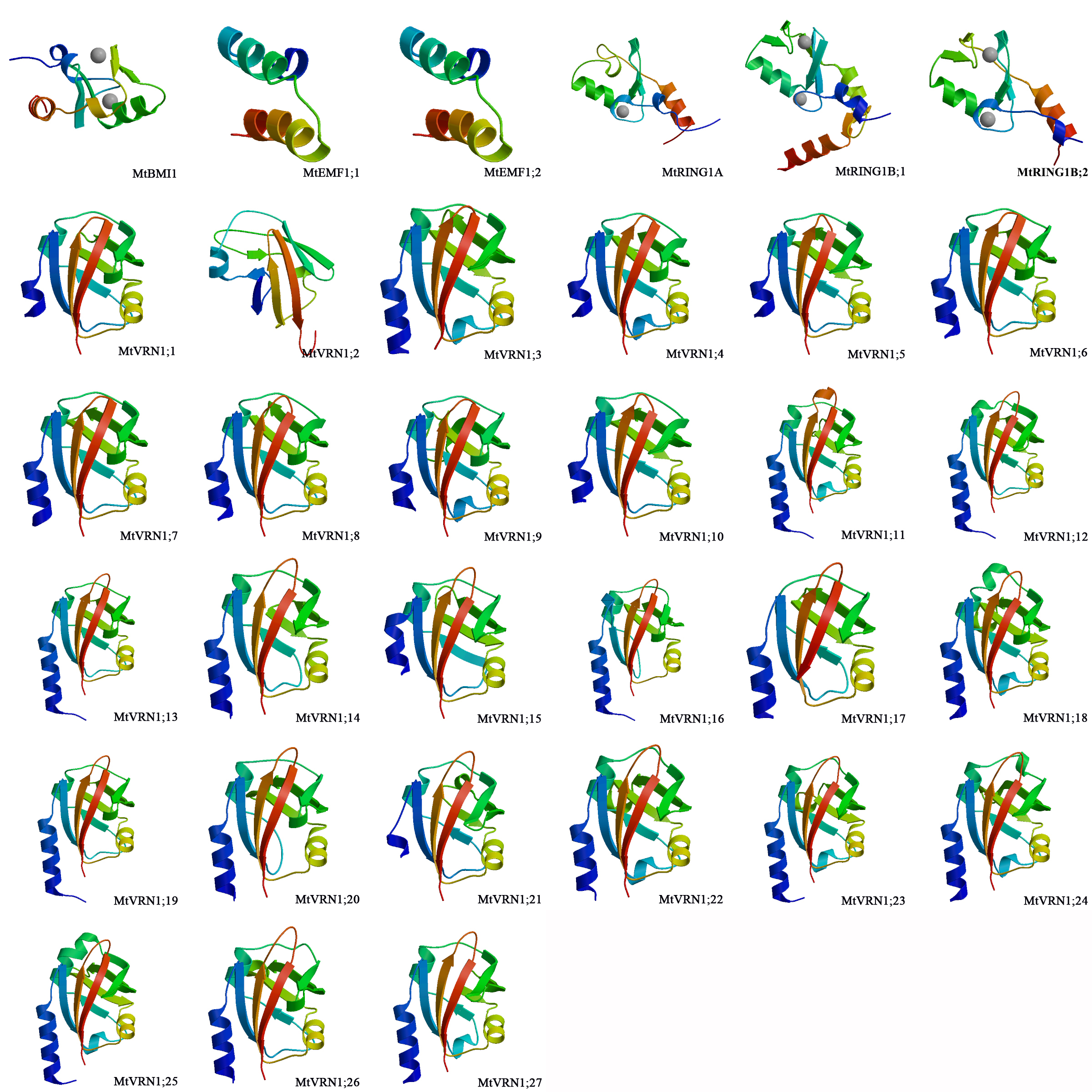

Supplement: Supplementary file 1 [file ijms-22-07537-s001.zip › Supplemental Figure S1 The prediction of tertiary structure of PRC1 proteins in M. truncatula.jpg]

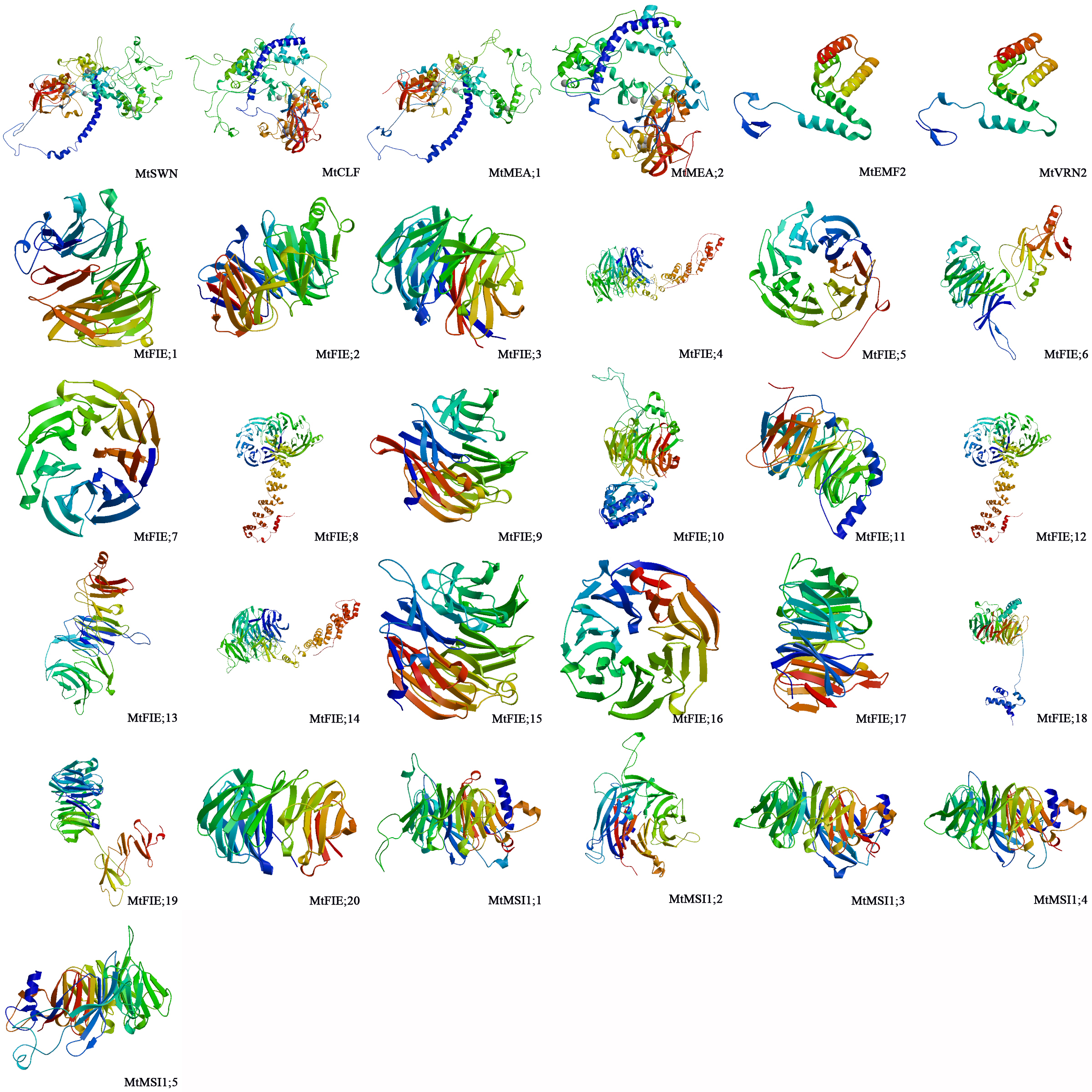

Supplement: Supplementary file 1 [file ijms-22-07537-s001.zip › Supplemental Figure S2 The prediction of tertiary structure of PRC2 proteins in M. truncatula.jpg]

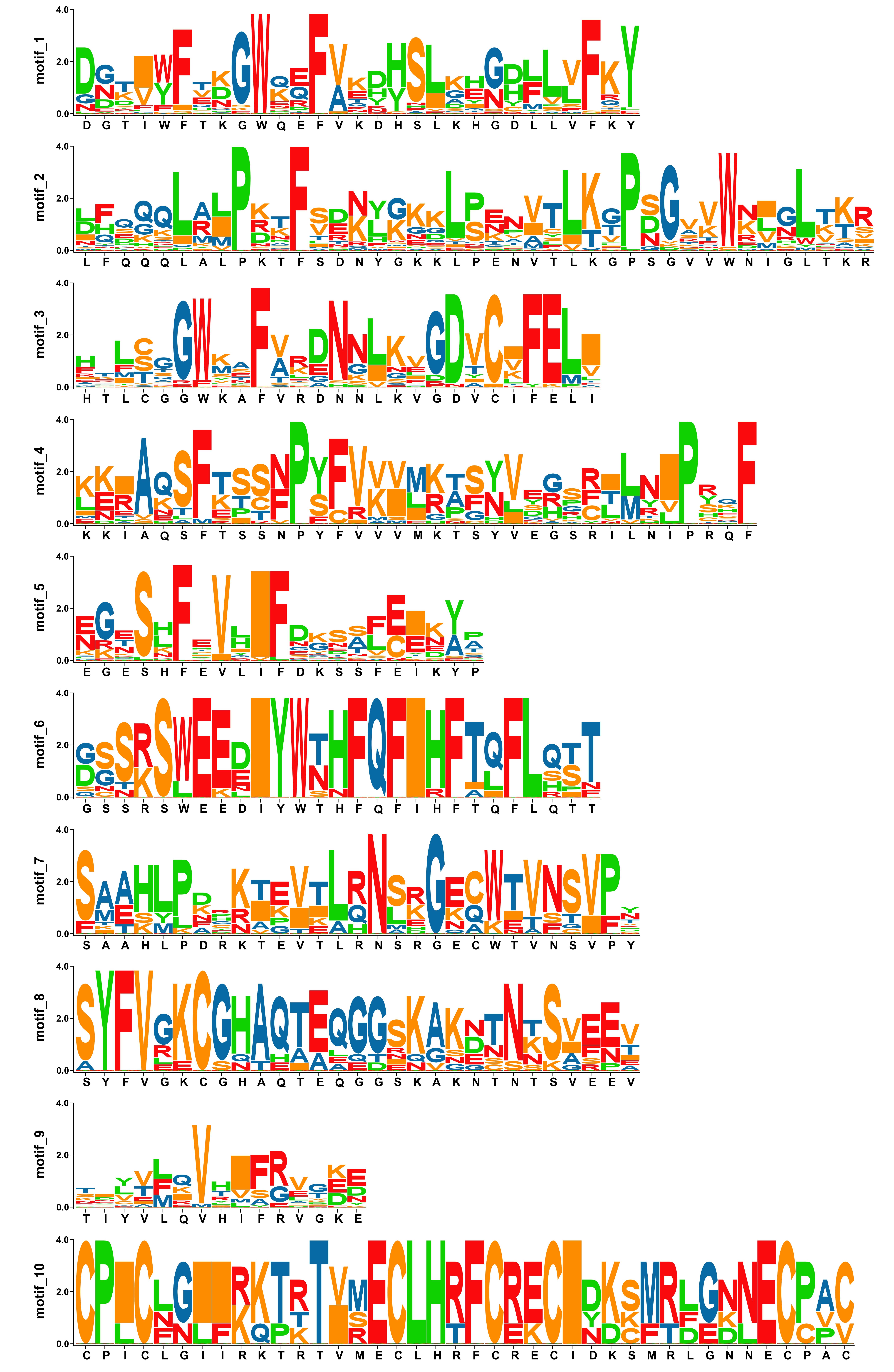

Supplement: Supplementary file 1 [file ijms-22-07537-s001.zip › Supplemental Figure S3 The motif logo analysis of PRC1.jpg]

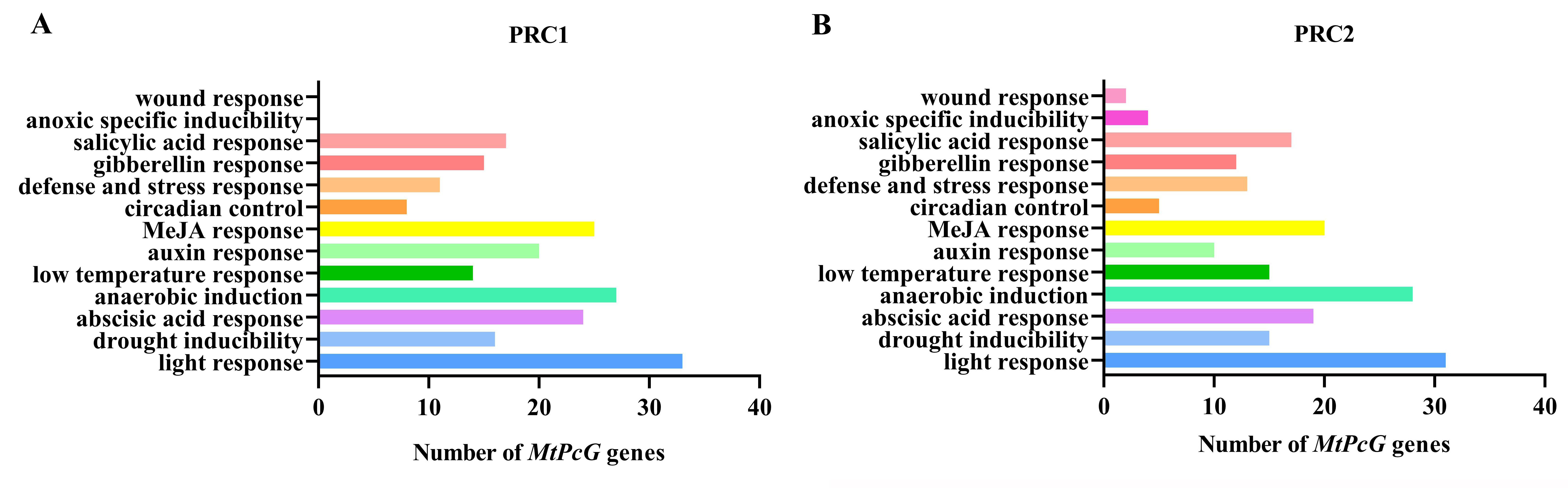

Supplement: Supplementary file 1 [file ijms-22-07537-s001.zip › Supplemental Figure S4 Statistics of cis-acting elements of PRC1 (A) and PRC2 (B).jpg]

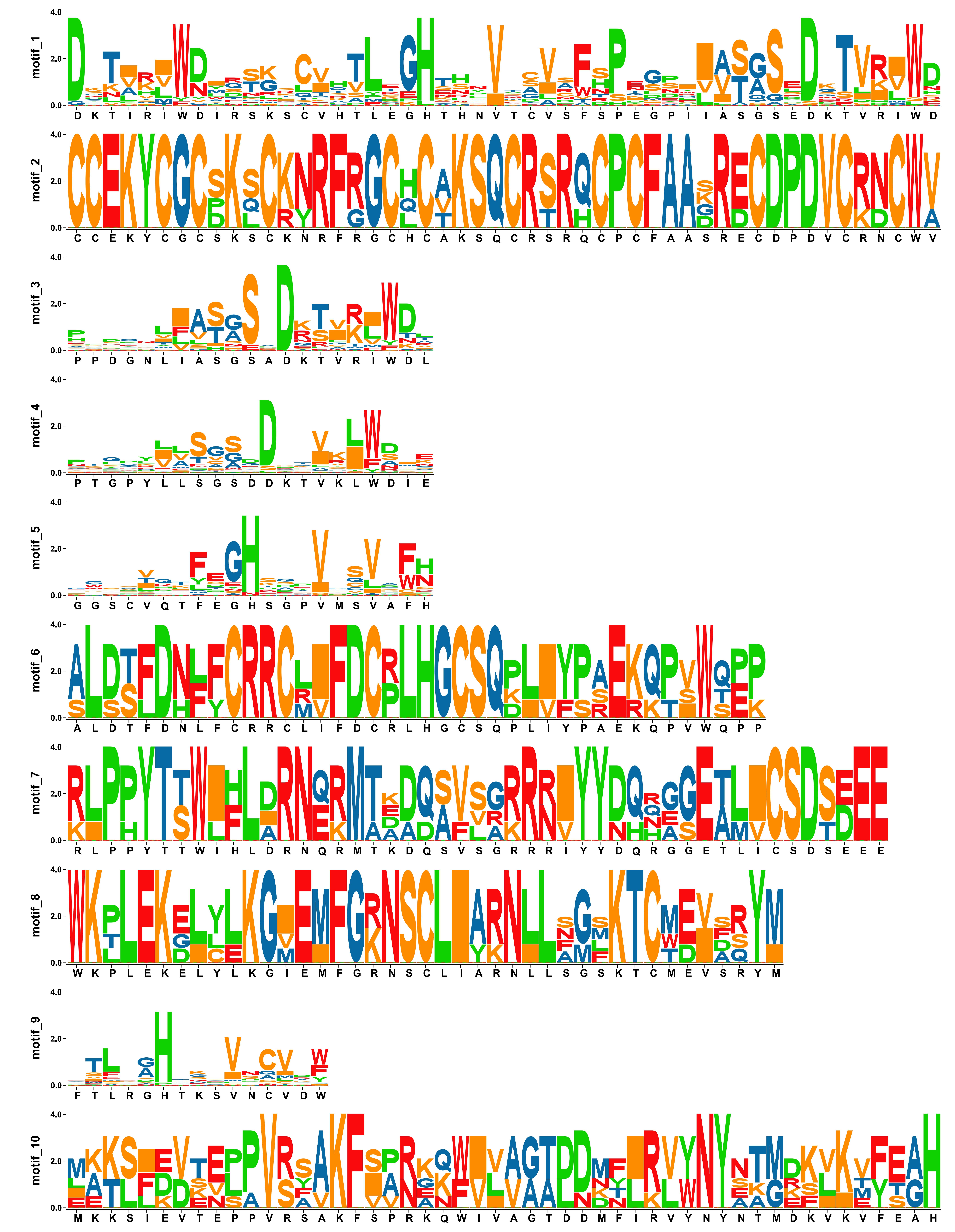

Supplement: Supplementary file 1 [file ijms-22-07537-s001.zip › Supplemental Figure S5 The motif logo analysis of PRC2.jpg]

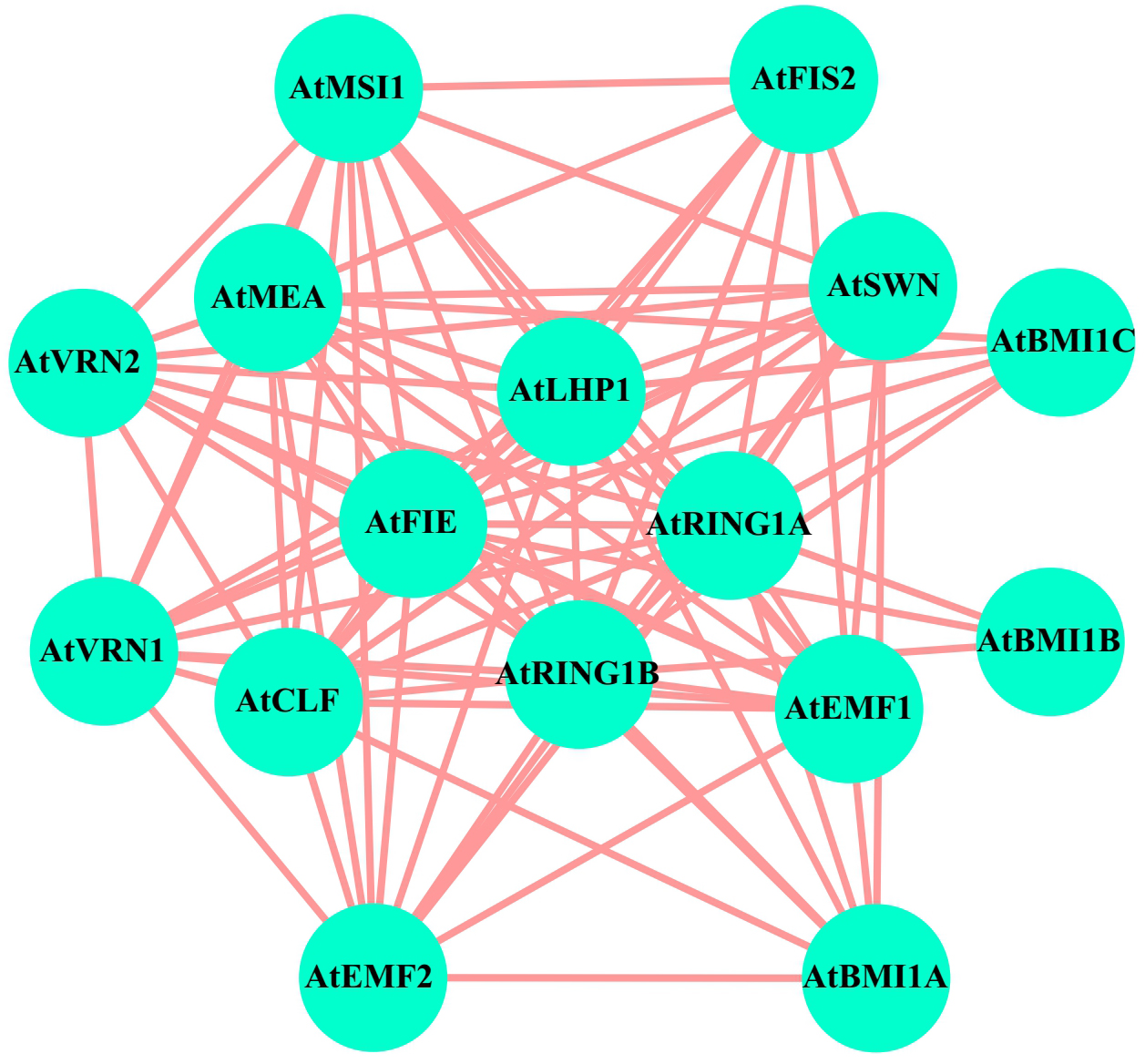

Supplement: Supplementary file 1 [file ijms-22-07537-s001.zip › Supplemental Figure S6 Prediction of protein–protein interaction among PcG members in A. thaliana.jpg]

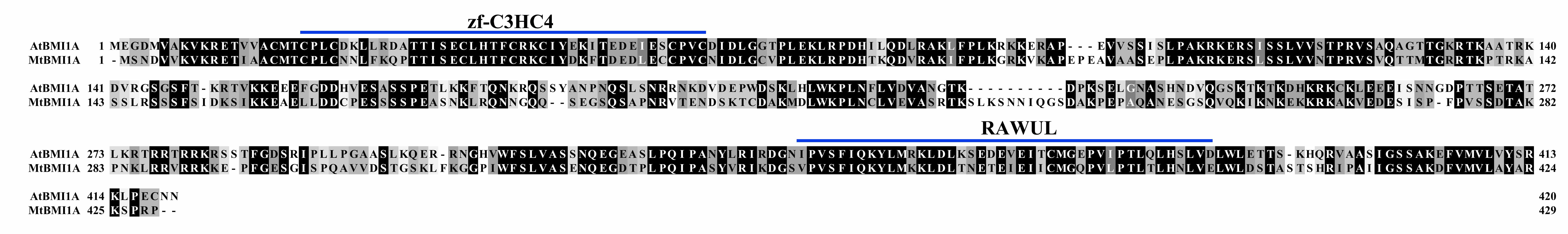

Supplement: Supplementary file 1 [file ijms-22-07537-s001.zip › Supplemental Figure S7–1 Multiple sequence alignment of BMI1 proteins between M. truncatula and A. thaliana.jpg]

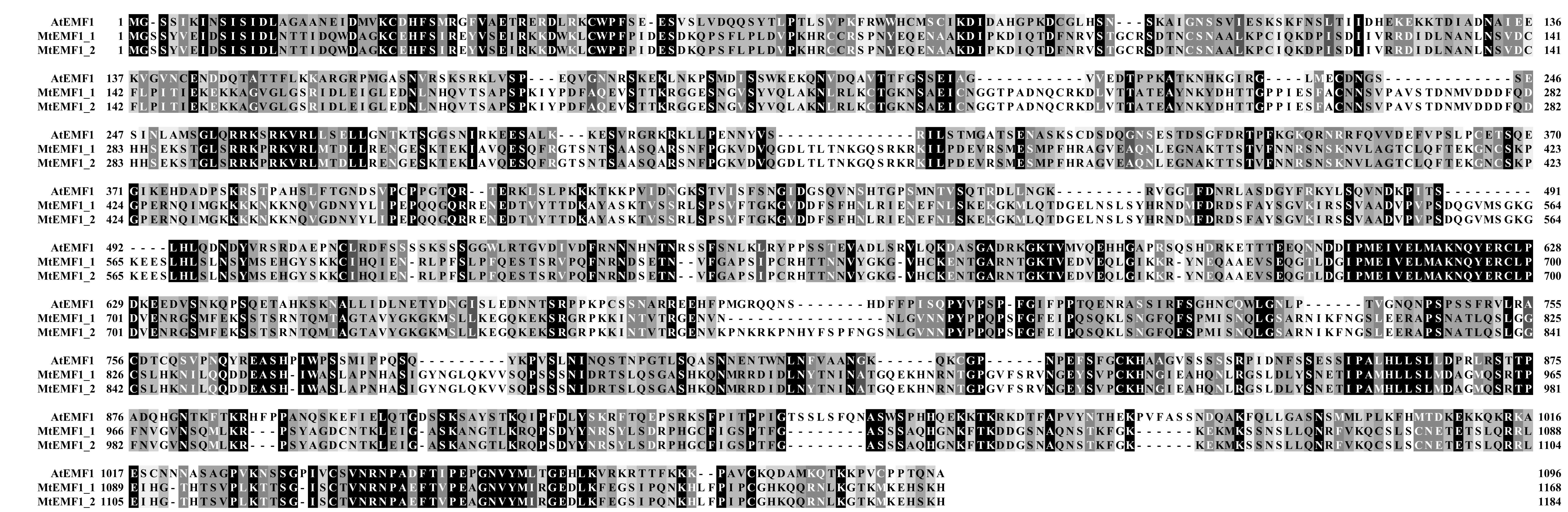

Supplement: Supplementary file 1 [file ijms-22-07537-s001.zip › Supplemental Figure S7–2 Multiple sequence alignment of EMF1 proteins between M. truncatula and A. thaliana.jpg]

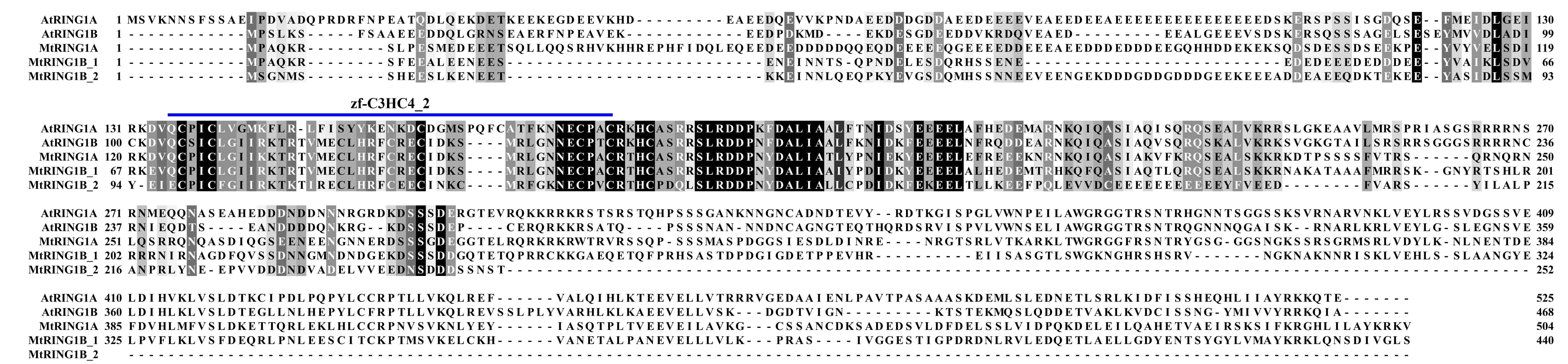

Supplement: Supplementary file 1 [file ijms-22-07537-s001.zip › Supplemental Figure S7–3 Multiple sequence alignment of RING1 proteins between M. truncatula and A. thaliana.jpg]

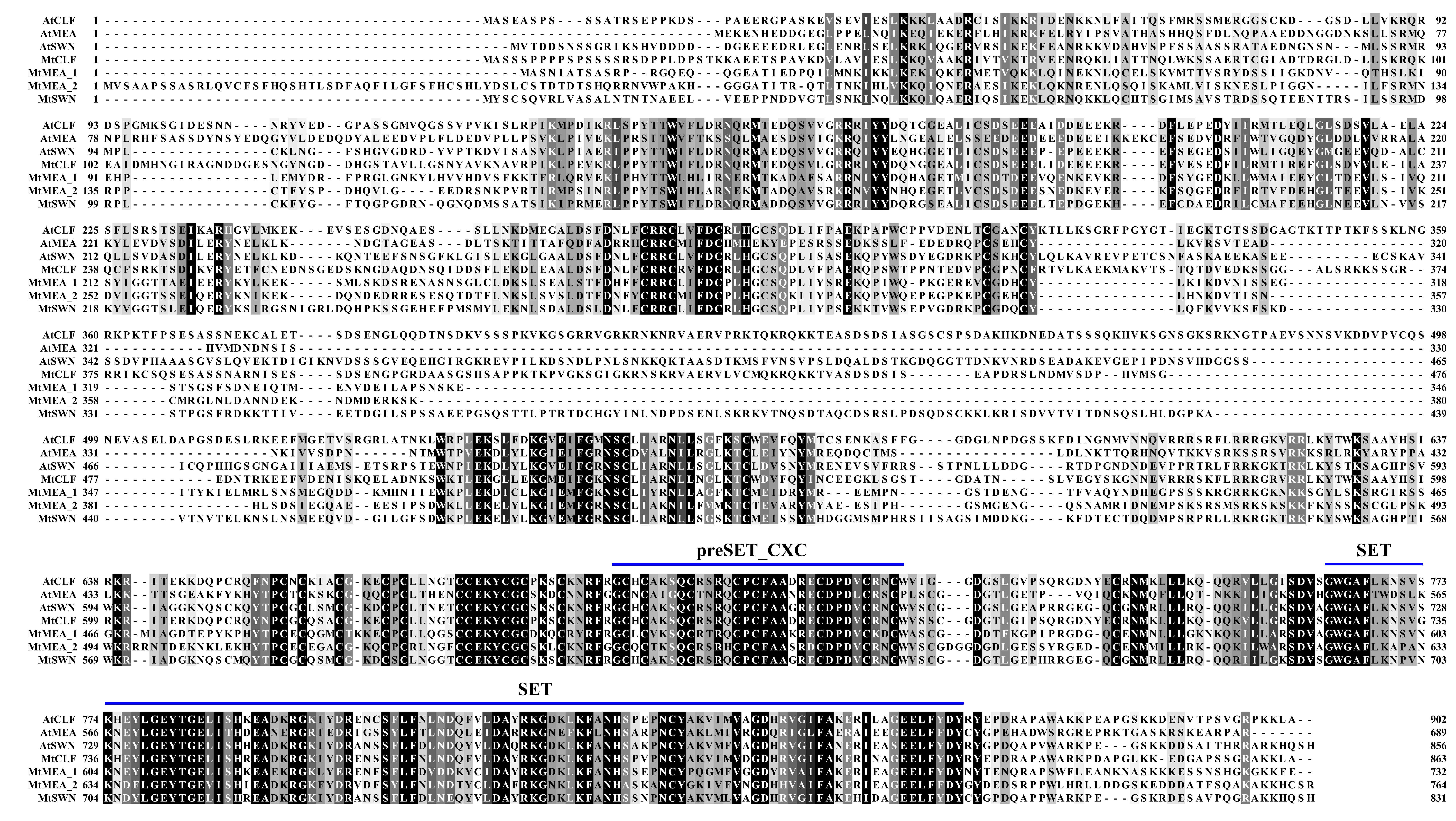

Supplement: Supplementary file 1 [file ijms-22-07537-s001.zip › Supplemental Figure S7–5 Multiple sequence alignment of SWN, CLF and MEA proteins between M. truncatula and A. thaliana.jpg]

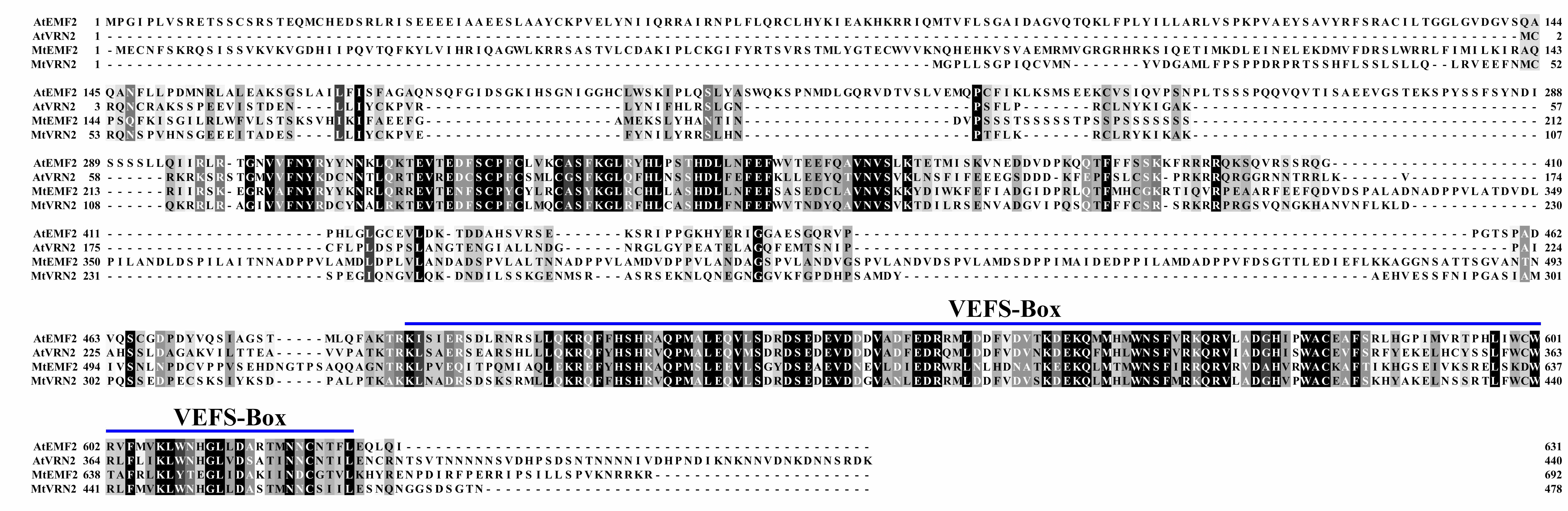

Supplement: Supplementary file 1 [file ijms-22-07537-s001.zip › Supplemental Figure S7–6 Multiple sequence alignment of EMF2 and VRN2 proteins between M. truncatula and A. thaliana.jpg]

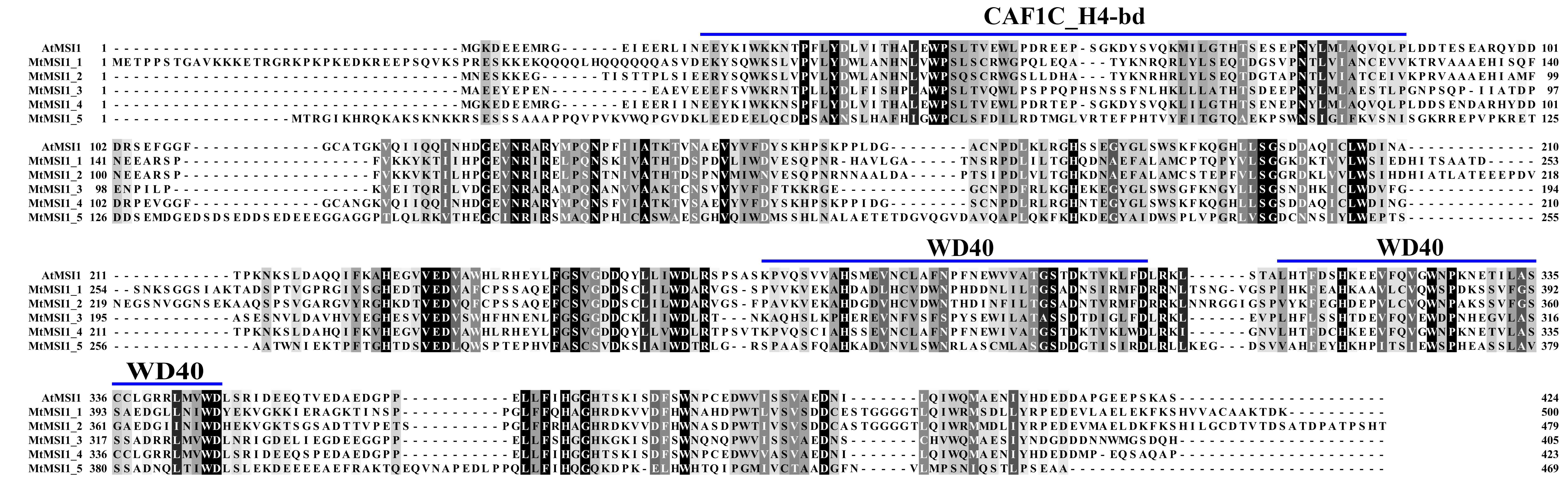

Supplement: Supplementary file 1 [file ijms-22-07537-s001.zip › Supplemental Figure S7–8 Multiple sequence alignment of MSI1 proteins between M. truncatula and A. thaliana.jpg]

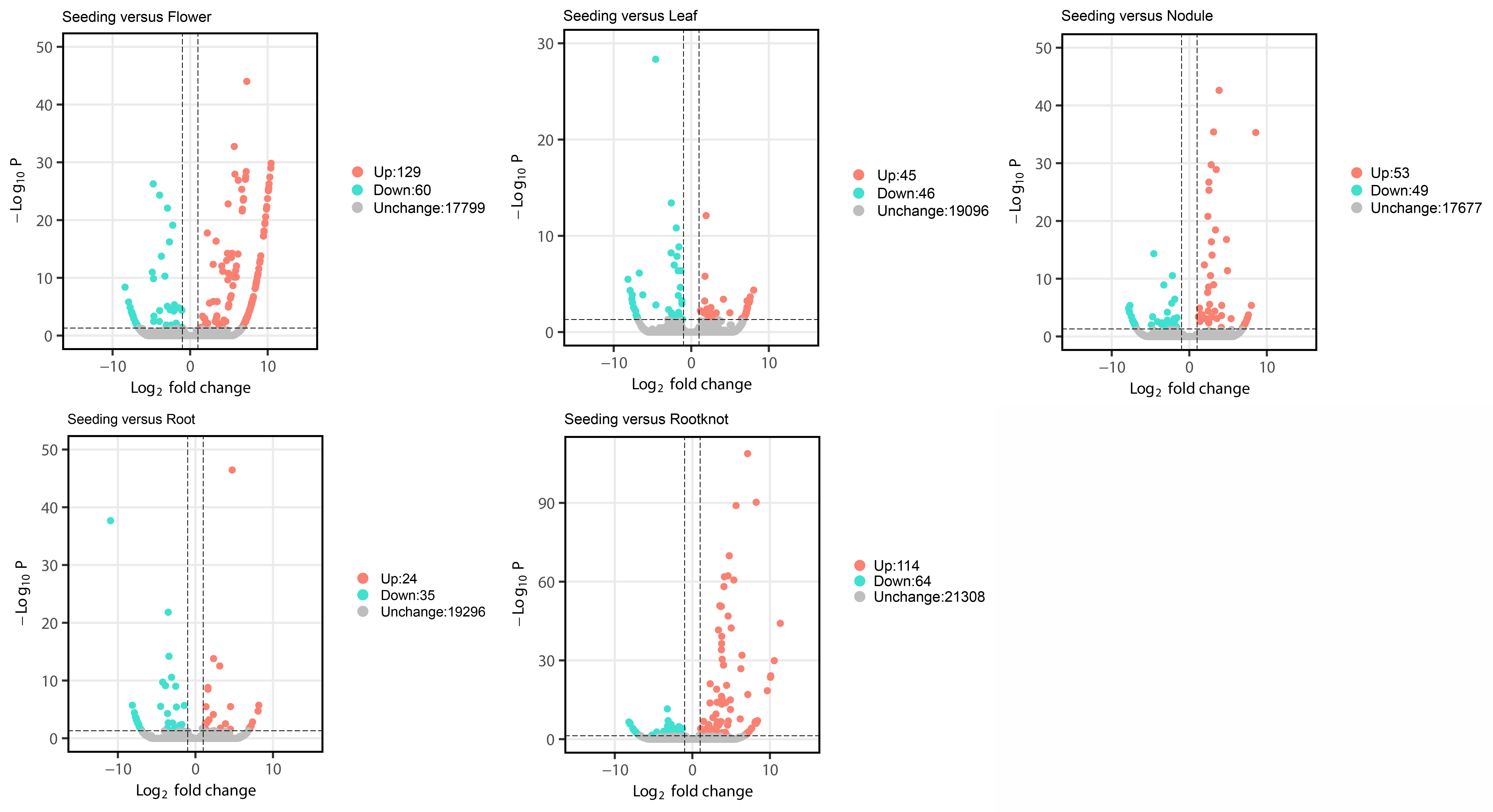

Supplement: Supplementary file 1 [file ijms-22-07537-s001.zip › Supplemental Figure S8 Identification of differentially expressed genes of PcG members based on RNA-seq data in different tissues.jpg]
